# Generating and sustaining long-lived spin states in ^15^N,^15^N′-azobenzene

**DOI:** 10.1038/s41598-019-56734-y

**Published:** 2019-12-27

**Authors:** Kirill F. Sheberstov, Hans-Martin Vieth, Herbert Zimmermann, Bogdan A. Rodin, Konstantin L. Ivanov, Alexey S. Kiryutin, Alexandra V. Yurkovskaya

**Affiliations:** 1 International Tomography Center SB RAS, Novosibirsk, 630090 Russia; 2 Helmholtz-Institut Mainz, Johannes Gutenberg-Universität, 55099 Mainz, Germany; 3 Freie Universität Berlin, 14195 Berlin, Germany; 4 Department of Biomolecular Mechanisms, Max-Planck-Institut für Medizinische Forschung, 69120 Heidelberg, Germany; 5 Novosibirsk State University, Novosibirsk, 630090 Russia

**Keywords:** Chemical physics, Chemical physics, Chemistry

## Abstract

Long-Lived spin States (LLSs) hold a great promise for sustaining non-thermal spin order and investigating various slow processes by Nuclear Magnetic Resonance (NMR) spectroscopy. Of special interest for such application are molecules containing nearly equivalent magnetic nuclei, which possess LLSs even at high magnetic fields. In this work, we report an LLS in *trans-*^15^N,^15^N′-azobenzene. The singlet state of the ^15^N spin pair exhibits a long-lived character. We solve the challenging problem of generating and detecting this LLS and further increase the LLS population by converting the much higher magnetization of protons into the ^15^N singlet spin order. As far as the longevity of this spin order is concerned, various schemes have been tested for sustaining the LLS. Lifetimes of 17 minutes have been achieved at 16.4 T, a value about 250 times longer than the longitudinal relaxation time of ^15^N in this magnetic field. We believe that such extended relaxation times, along with the photochromic properties of azobenzene, which changes conformation upon light irradiation and can be hyperpolarized by using parahydrogen, are promising for designing new experiments with photo-switchable long-lived hyperpolarization.

## Introduction

Long-Lived spin States (LLSs)^1–3^ represent an important emerging methodology in Nuclear Magnetic Resonance (NMR), as they allow one to investigate various slow processes^4–6^ and to sustain spin hyperpolarization^7–17^, thus providing enormous NMR signal enhancements^18,19^. In most cases experiments reported to date, an LLS corresponding to the singlet order of a pair of spins-1/2 is investigated. By singlet order we mean the population imbalance between the singlet state and triplet states. Such a population imbalance is immune to the mutual dipolar relaxation of two spins, which typically gives the main contribution to relaxation, in particular for protons, and relaxes only due to less efficient mechanisms. Consequently, the singlet order lifetime, *T*_*S*_, can be much longer than the *T*_1_-relaxation time of the spin pair, which corresponds to the relaxation time of the longitudinal spin magnetization. Previous works report very large *T*_*S*_/*T*_1_ ratios and *T*_*S*_ relaxation times reaching minutes for ^1^H spins^20,21^, tens of minutes^22,23^ or even more than an hour^24^ for pairs of ^15^N or ^13^C spins. All these examples of extremely long singlet state lifetimes have been found at low magnetic fields. At magnetic fields typical for detection for most nuclei (e.g. ^13^C, ^15^N, ^19^F) Chemical Shift Anisotropy (CSA) gives the dominant contribution to relaxation. In this situation, extended lifetime of the singlet order is expected when the CSA tensors of the spin pair have the same principal axes systems and the same principal values^25^.

Although most examples of LLS are limited to pairs of coupled spins, it is of interest to extend the LLS methodology to  multispin systems. Examples like this have been previously reported, which are given by three or four coupled spins, in which there is a dominant spin-spin coupling that isolates the singlet state of a given pair from the triplet states (“localized” singlet states due to J-stabilization mechanism)^26–28^, and by symmetric molecules with groups of chemically equivalent but magnetically non-equivalent spins^11,12,17,29–31^. One of the systems of the latter kind is given by azobenzene (ABZ) labelled with two ^15^N-spins, which is the focus of this work.

**Figure 1:**
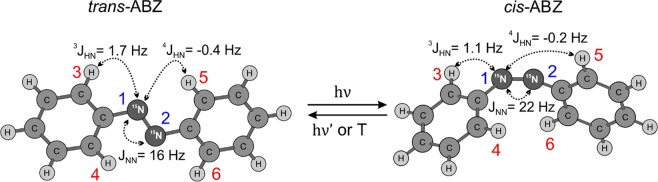
Structure of *trans-* and *cis-*^15^N, ^15^N′*-*azobenzene and *trans-* to *cis-*conversion reaction; we specify the numbering of an ${\rm{AA}}\text{'}{{\rm{X}}}_{2}{{\rm{X}}}_{2}^{\text{'}}$ spin system and relevant *J*-couplings exploited to enrich the singlet order of the ^15^N spin pair.

ABZ exists in two isomeric forms, *trans*-ABZ and *cis*-ABZ, see Fig. 1. At room temperature, ABZ exists in the *trans*-form; switching between the two isomers can be performed by using light excitation^32^. This photochromic property of ABZ makes it a promising probe for conformational control, in particular, of biomolecules^33^. Only in *trans-*ABZ the singlet order of the ^15^N pair is long-lived^34^: the molecule has an inversion symmetry, which protects the LLS against relaxation driven by CSA and mutual dipole-dipole interaction of the ^15^N spins, while *cis*-ABZ does not have any geometric symmetries because of the steric repulsion of the two phenyl rings. This effect is similar to a previous observation in the derivative of ^13^C_2_-fumarate: dipole-dipole interactions, which are not symmetric under spin permutation destroy the LLS in the *cis-* isomer, whereas for *trans-* geometry they are symmetric and do not cause relaxation of the LLS^35^. On the other hand, c*is-*ABZ is the isomer that can be polarized by using a parahydrogen-based method^36^: this paves the way to long-lived spin hyperpolarization, which can be implemented by combining experiments on *cis*-ABZ with parahydrogen, controllable photo-switching and storage of the nuclear hyperpolarization in the form of an LLS of *trans-*ABZ nuclear spins. Such experiments are underway in our laboratory.

The ABZ molecule comprises chemically equivalent nuclei, so that conversion of spin magnetization to singlet order (generation of the LLS) and backward conversion (required for readout of the LLS) are possible only due to the weak magnetic non-equivalence of these nuclei. Such magnetic non-equivalence arises from the difference of spin-spin interactions of the two ^15^N labels with different protons in the molecule. The conversion requires special NMR methods like magnetization-to-singlet (M2S)^37,38^ and Spin-Locking Induced Crossing (SLIC)^39^ or Adiabatic Passage Spin Order Conversion (APSOC)^40^ and adiabatic SLIC methods (adSLIC)^41,42^. It is worth noting that the J-coupling network of ^15^N_2_-ABZ is analogous to ^13^C_2_-1,2-diphenylacetylene, for which an attractive opportunity of generating and detecting an LLS by NMR excitation of protons as well as carbons has been discovered^17,31^. Here we analyze these two types of conversion between spin magnetization and LLS and perform experiments with the LLS by applying pulses on the nitrogen as well as on the proton channel. We analyze in detail the conversion efficiency: while for two spins-1/2 it is well-known that the highest possible single-step conversion efficiency from magnetization to the singlet order and backwards is equal to 2/3^43^, for multi-spin systems, like that of ABZ, the upper limit for spin order conversion has not been established so far. To perform such an analysis and to model experiments with singlet order, we consider ABZ as a simplified ${\rm{AA}}\text{'}{{\rm{X}}}_{2}{{\rm{X}}}_{2}^{{\prime} }$ system, which contains two ^15^N nuclei and four *ortho-*protons, highlighted in Fig. 1. The calculated maximal intensities of the LLS component correspond to 26% of ^15^N magnetization and 13% of ^1^H magnetization in such a simplified spin system. Analysis shows that these values are achieved only using adiabatic methods, whereas the amplitude of LLS produced by resonance techniques, such as SLIC or M2S, is limited by 16% of ^15^N magnetization and 8% of ^1^H magnetization.

First, we perform experiments using the SLIC method. The main benefit of SLIC compared to other techniques is that it has the shortest duration. Also it is easy to implement. SLIC allows good efficiency of singlet-triplet transfer in near-equivalent spin pairs and finds application for transfer of the parahydrogen singlet order to magnetization^44–46^. To achieve efficient spin order conversion, we make use of adiabatic NMR pulses (applied in the manner of adSLIC or APSOC) and optimize the pulse envelopes to obtain “constant adiabaticity” radiofrequency (RF) pulses^42,47^.

It is important to note that all methods (SLIC, adSLIC, APSOC) make use of the weak magnetic non-equivalence of nuclei in ABZ rather than chemical non-equivalence (which gives rise to a chemical shift difference of the involved spins). Consequently, the proposed schemes are applicable to generate and detect the LLS at any magnetic field.

Due to the fact that the ^15^N spins in *trans*-ABZ are nearly equivalent the triplet-singlet leakage^48^ rate is low: we previously have demonstrated that the LLS in *trans-*ABZ can be sustained even without applying spin-locking RF-fields with the LLS lifetime of up to 2 minutes at high magnetic fields (9.4 and 16.4 T)^34^. Here we show that the lifetime of the LLS in *trans-*ABZ can be increased further, reaching 17 minutes under spin-locking at the high field of the NMR spectrometer.

## Theory

In ABZ, there are two chemically equivalent ^15^N nuclei, which are, however, magnetically non-equivalent, since they have different *J*-couplings to the protons of the phenyl rings. All proton-nitrogen *J*-couplings are averaged because of the fast internal rotations of the phenyl rings around the CN bonds, which results in groups of identical J-couplings: ${}^{4}{J}_{15}={}^{4}{J}_{16}={}^{4}{J}_{23}={}^{4}{J}_{24}={}^{4}{J}_{HN}$ and ${}^{3}{J}_{13}={}^{3}{J}_{14}={}^{3}{J}_{25}={}^{3}{J}_{26}={}^{3}{J}_{HN}$; these couplings are also presented in Fig. 1. Magnetic non-equivalence can be considered such that for specific states of protons the two ^15^N-labels experience slightly different local magnetic fields. More accurately, these local fields are the same for some proton states, causing effective A_2_-type sub-spectra of the two ^15^N nuclei, being different for some other states, resulting in effective AB-type sub-spectra in the ^15^N NMR spectrum. Thus, an effective difference in the NMR frequencies of the nitrogen spins can be introduced here, which is proportional to the differences $\Delta J={}^{3}J_{HN}-{}^{4}J_{HN}$ of the couplings to the protons in the nearby and the remote phenyl ring. The ${J}_{NN}$ coupling is always much larger than $\Delta J$, giving rise to strong coupling between the ^15^N spins. Hence, the pair of the ^15^N spins constitutes either an A_2_ system or a pair of nearly equivalent spins $AA{\prime}$. In such pairs of nearly equivalent spins the LLSs can be sustained^34^ even without suppressing the non-symmetric part of the coherent interaction (originating from the $\Delta J$ term in the Hamiltonian, see Supplementary Information), which is commonly done by applying spin-locking.

To model the spin dynamics of creation of the singlet order in *trans-*ABZ, we consider the simplified 6-spin ${A{\rm{A}}{\prime} X}_{2}{{\rm{X}}}_{2}^{{\prime} }$ system where A and A′ correspond to the ^15^N nuclei and X and X′ stand for *ortho-*protons (Fig. 1). Reduction of the spin system to only 6 nuclei is clearly a simplification; nonetheless, such a simplification was successfully used before to model LLSs in ^13^C_2_-1,2-diphenylacetylene^17,31^. The *J*-coupling constants have been reliably determined in our previous work^34^ reporting a detailed analysis of ^1^H and ^15^N NMR spectra of *cis*- and *trans*-ABZ. Note, that *J-*couplings between protons from different phenyl rings are negligibly small, as they do not appear in the high resolution ^15^N and ^1^H spectra of ABZ, while simulated spectra with their values set to *zero* fit the NMR lineshapes very well^34^. The LLS of interest in this system is the singlet state of the two ^15^N spins: this state does not relax due to the dipole-dipole interaction of the nitrogen spins and the symmetric part of the CSA interaction, but only through less efficient mechanisms. The main cause of LLS relaxation is given by the dipole-dipole interactions of the ^15^N spins with protons. However, these interactions are small, as is apparent from the absence of cross-relaxation in the molecule (absence of the steady-state ^1^H-^15^N NOE between *ortho-*protons and nitrogen spins, not shown here). As has already been mentioned, the LLS can be sustained in the absence of spin-locking. However, as we show below, spin-locking significantly increases the LLS lifetime, revealing that singlet-triplet leakage is an essential relaxation mechanism here.

**Figure 2:**
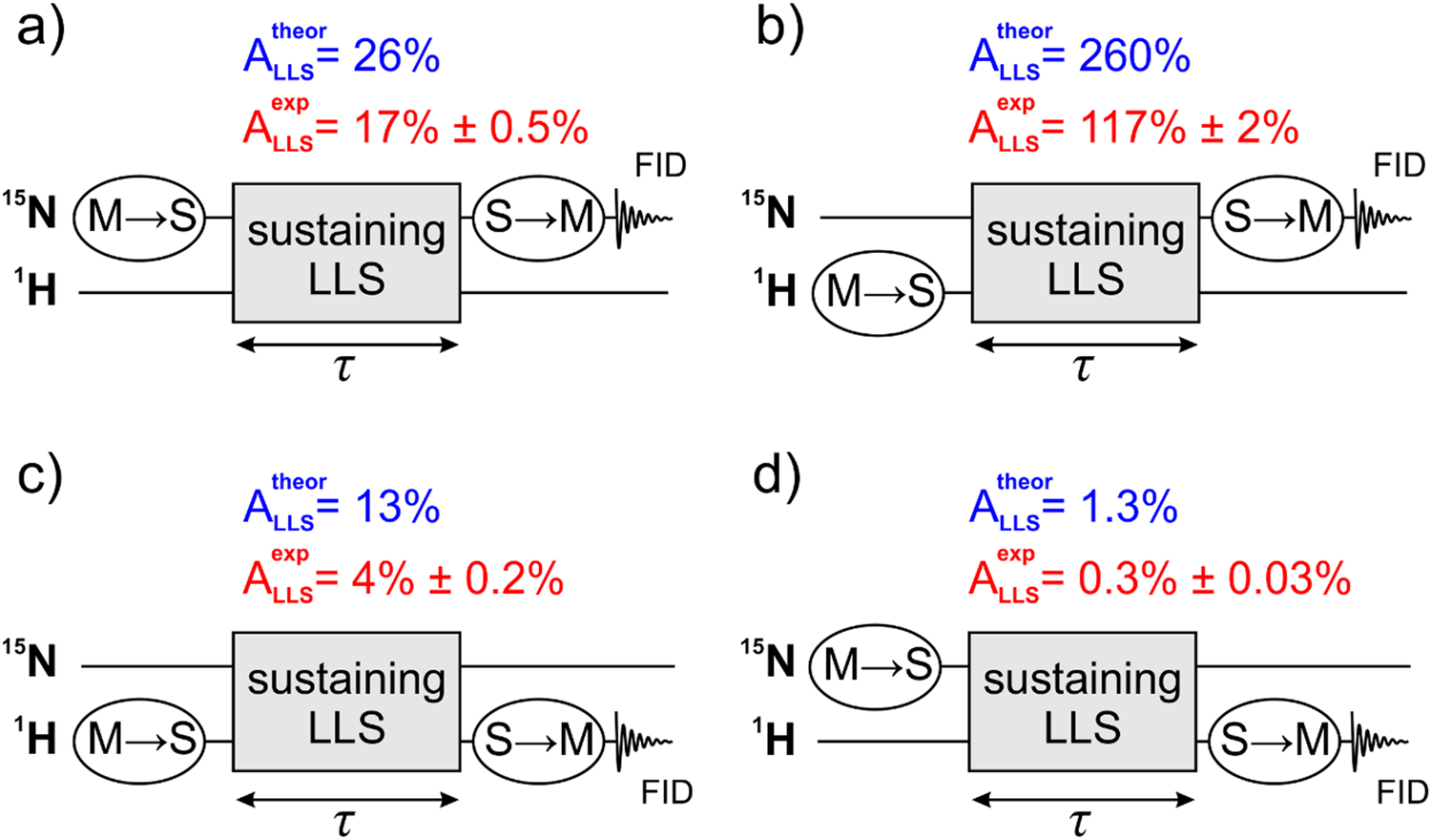
Experimental schemes (**a**–**d**) proposed to access and detect the LLS using three blocks: (i) magnetization-to-singlet (M → S) conversion, (ii) sustaining an LLS and (iii) singlet-to-magnetization (S → M) conversion followed by detection of the FID signal. Magnetization of both nitrogen or protons can be used to generate the LLS by an M → S block, likewise, the LLS can be converted into the magnetization of either kind of nuclei by an S → M block. In some experiments we also used filters to observe only the long-lived spin order. For further detail, see text. We specify the theoretically possible (for the model AA′X_2_X′_2_ spin system) and the experimentally achieved amplitudes of the LLS-derived signals for all schemes. The experimental values (and errors) are taken from experiments performed with spin-locking to sustain LLS (see below). The values are given in percent of the thermal signal on the corresponding free induction decay (FID) detection channel.

One more feature of this spin system, which has been reported before^17,35^, is that one can populate the LLS not only using the nitrogen spin magnetization, but also using the proton spin magnetization. Likewise, readout of the LLS can be performed by the transfer to either nitrogen or proton magnetization. Hence, the experiments were carried out as shown in Fig. 2. In order to distinguish between different cases we define the blocks of conversion of the magnetization-to-singlet (M → S) and the singlet-to-magnetization (S → M) and assign them to the corresponding NMR channels (proton channel or nitrogen channel), as shown in Fig. 2. In each experiment, the LLS is sustained during a variable time period *τ*. After the S → M block transverse magnetization is generated, which gives rise to the Free Induction Decay (FID) signal; the Fourier transform of the FID signal gives the NMR spectrum. In ABZ it is possible to sustain the LLS either without spin-locking, or by applying a spin-locking RF-field on the proton or nitrogen channel or on both channels, as discussed below. We also used filters to detect only the LLS-derived signal and suppress other signals in the NMR spectrum. These methods are the singlet order selection filter^49,50^ and the so-called T_00_-filter^51^; details of them are discussed in section 6, “Methods”.

**Figure 3:**
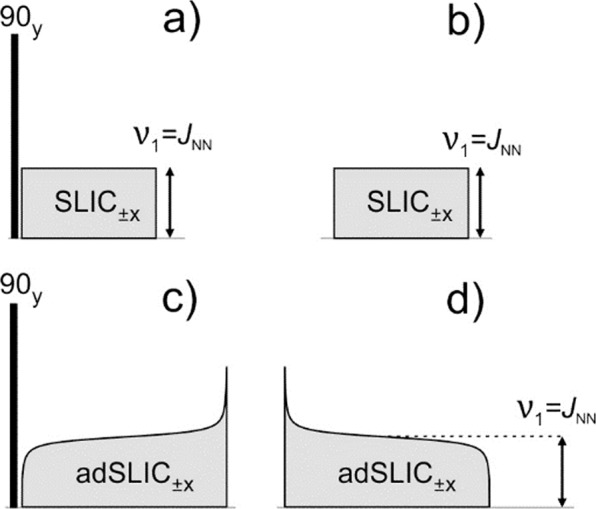
NMR methods used to perform M → S and S → M conversion: the SLIC and adSLIC methods are exploited. Here (**a**,**b**) show the pulses for the SLIC method for M → S and S → M conversion, respectively. Similarly, (**c**,**d**) show the conversion scheme for the adiabatic adSLIC method. For nearly equivalent spins it does not matter whether the amplitude of the adiabatic RF field increases or decreases. All these spin order conversion blocks can be applied on both ^1^H and ^15^N channels, as shown in Fig. 2.

Figure 3 shows the NMR pulse sequences used for generating (M → S) and detecting (S → M) the LLS. Here we consider two methods: traditional SLIC^39^ and adiabatic adSLIC^41,42^. In SLIC, transverse magnetization is generated by a 90-degree pulse; subsequently, a spin-locking field is applied. The idea of the method is that transverse magnetization is parallel to the effective field in the rotating frame with the consequence that it is “locked” to the field; however, when the field strength is matched to the *J*_*NN*_ coupling, M → S conversion is taking place. The S → M conversion is driven by another SLIC pulse: at the end of the pulse transverse magnetization is generated and detected. The duration of a SLIC pulse must be carefully chosen as discussed in a previous paper^39^.

In adSLIC, instead of a rectangular RF-pulse a shaped pulse with variable amplitude is used in order to carry out a slow passage through the Level Anti-Crossing (LAC) region. Hence, the M → S block consists of a 90-degree pulse and a shaped adSLIC pulse with increased RF-amplitude. The S → M block is given by a shaped RF-pulse with decreased amplitude. For nearly equivalent spins both adiabatic increase and decrease of the RF-amplitude perform the required conversion. The strategy for optimization of the pulse envelopes is discussed in the section 6, “Methods”.

In addition to SLIC, we also applied the APSOC method, which gives a conversion efficiency comparable to that achieved in adSLIC. However, to keep the description simpler, we discuss these results only in Supplementary Information. Supplementary Information additionally contains sections on detailed analysis of the coherent spin dynamics, which takes place when ^15^N singlet order is created on ^15^N and ^1^H channels. Furthermore, we give a derivation of the maximal amplitudes of the LLS signal in case of a model ${\rm{AA}}\text{'}{{\rm{X}}}_{2}{{\rm{X}}}_{2}^{{\prime} }$ spin system. The results of these analyses are summarized in Fig. 2.

## Results and Discussion

**Figure 4:**
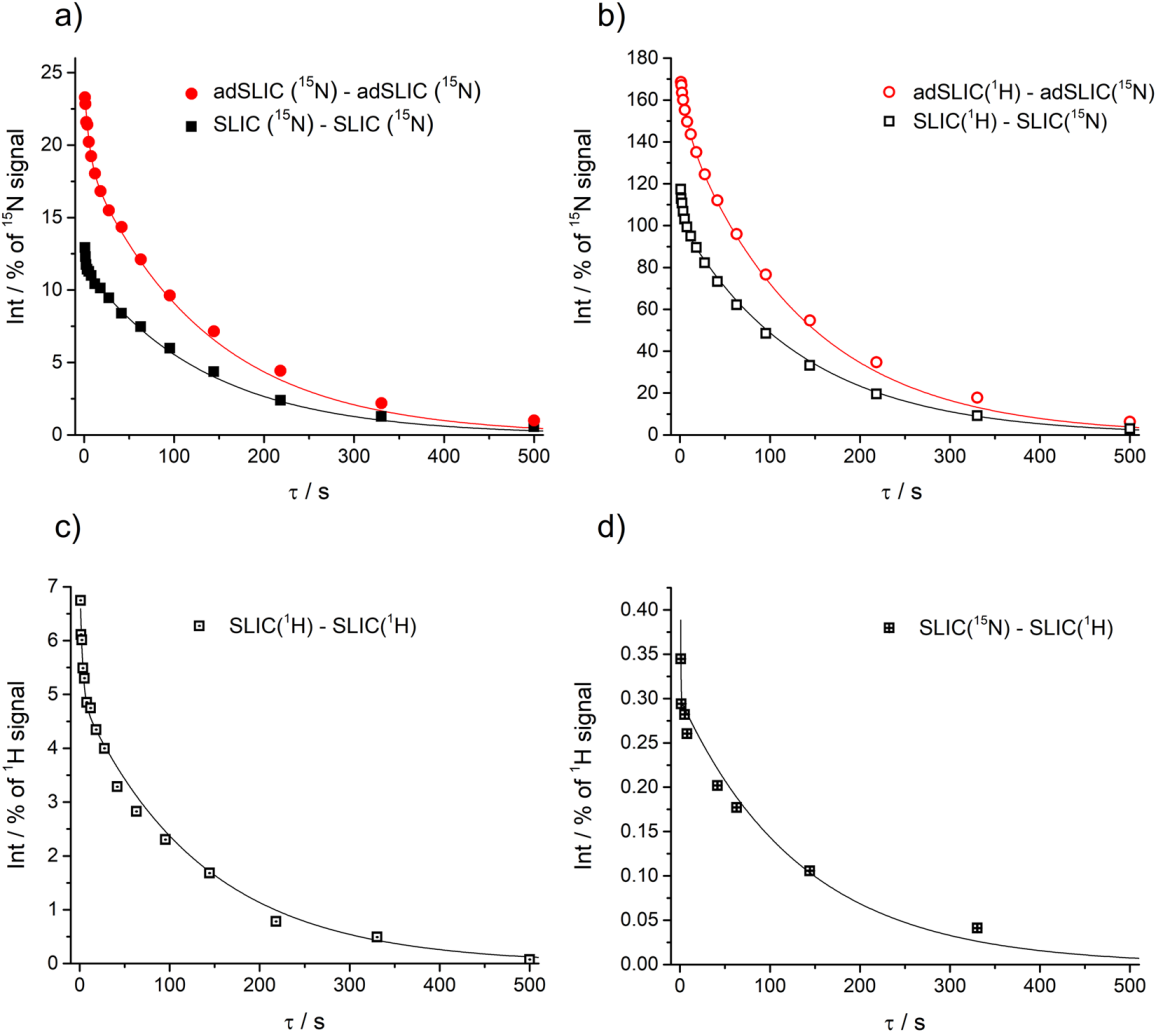
Relaxation traces obtained in experiments without using spin-locking for sustaining the LLS. The M → S and S → M conversion performed on the following channels: ^15^N-^15^N (**a**), ^1^H-^15^N (**b**), ^1^H-^1^H (**c**) and ^15^N-^1^H (**d**). Spin order conversion has been done using SLIC and adSLIC (the specific method is indicated for each subplot). All relaxation traces were fitted using a common lifetime ${{T}}_{{LLS}}=135$ s of the long-lived component with the amplitude *A*_*LLS*_ equal to (**a**) 11.3% for SLIC and 18.4% for adSLIC, (**b**) 101% for SLIC and 147% for adSLIC, (**c**) 4.9%, (**d**) 0.3%. Parameters of each individual fitting are available in the Supplementary Information for experiments #1, #7 (**a**) #14, #15 (**b**), #19 (**c**), #22 (**d**).

We start from experiments without using spin-locking for sustaining the LLS and compare all four schemes for M → S → M conversion (see Fig. 2). The typical decay curves are shown in Fig. 4. The *τ*-dependence of the signal, i.e., the LLS relaxation curves, were fitted by bi-exponential decay functions:

1 $${I}({\tau })={{I}}_{0}+{{A}}_{{fast}}\exp \{-\frac{{\tau }}{{{T}}_{{f}}}\}+{{A}}_{{LLS}}\exp \{-\frac{{\tau }}{{{T}}_{{LLS}}}\}$$

Here *T*_*f*_ is the relaxation time for the “fast” component (triplet relaxation time) and *T*_*LLS*_ stands for the lifetime of the “slow” component, i.e., the LLS of interest. The coefficient *A*_*LLS*_ gives the intensity of the LLS component: when measured in units of thermal polarization it gives the M → S → M conversion efficiency.

Figure 4 clearly shows that the LLS lifetime does not depend on the type of scheme for generating and detecting the LLS, in agreement with the theoretical consideration that in all cases the same singlet order is created. For this reason, we fitted the decaying curves using the common relaxation time *T*_*LLS*_ = 135 s; the standard deviation from this value, as determined from fitting all relaxation traces with different *T*_*LLS*_ values, was found to be ±17 s. As far as the signal intensity is concerned, it is optimal to populate the singlet order from the 10-fold higher thermal ^1^H polarization: this gives rise to a *ca*. 10-fold enhancement of the long-living spin order and the resulting NMR signal. The LLS-derived signal is then even stronger than the ^15^N signal coming from the thermal polarization, reaching up to 147% of the thermal ^15^N signal (Fig. 4b). However, we are still not able to achieve the theoretically predicted values (upper limit).

The determined *A*_*LLS*_ values, which stand for the efficiency of generating the LLS, are closer to the theoretical values when the singlet order is generated and detected on the ^15^N channel by using adiabatic methods. The observed intensity of the LLS signal (18% of thermal magnetization) corresponds to efficiency of *ca*. 80% for the M → S and S → M conversion steps, considering simplified spin system ${\rm{AA}}\text{'}{{\rm{X}}}_{2}{{\rm{X}}}_{2}^{{\prime} }$ (detailed explanations are given in Supplementary Information). When the singlet order was generated by applying pulses on the ^1^Н channel and detected on the ^15^N channel we have found an efficiency of 147% (being still smaller than the expected 260%); for proton detection we obtained around 5% and 0.4% efficiencies (with 13% and 1.3% maximal theoretically allowed values, respectively). In addition to detrimental effects of the *B*_1_-field inhomogeneity and spin relaxation during the pulse, a possible factor for getting a lower conversion efficiency than expected from theory is that the actual spin system is more complex than the ${\rm{AA}}\text{'}{{\rm{X}}}_{2}{{\rm{X}}}_{2}^{\text{'}}$ system. For instance, interactions with the *para-*protons also cause magnetic non-equivalence of the ^15^N spins (the corresponding Δ*J* value is about 0.6 Hz). Hence, even the *para-*protons can affect the relevant singlet-triplet LACs, but this is not taken into consideration here.

One can clearly see that adiabatic methods work approximately 50% more efficiently in the cases where detection is performed on the ^15^N channel. The reason is that adiabatic methods are generally more robust^41,42,47^ to inaccuracies in setting the RF-field parameters (RF-amplitude and frequency) and inhomogeneity of the *B*_0_-field and RF-field. At the same time, in the case of ^1^H detection we could not acquire satisfactory data with adSLIC. The reason is, most likely, that adiabatic pulses are too long so that a substantial fraction of the thermal NMR signal is recovered during application of the pulses. As a consequence, it becomes difficult to extract and analyze the LLS-derived signal.

In the case of proton NMR detection, for the M(^1^H)→S →M(^1^H) scheme the LLS-derived signal corresponds to approximately 5% of the thermal proton polarization. However, one should note that the number of *ortho-*protons, building the ^1^H signal, is two times greater than the number of ^15^N spins, meaning that the efficiency of populating the LLS is comparable with that in the scheme M(^1^H)→S →M(^15^N). At the same time, proton NMR detection is more sensitive as compared to ^15^N-NMR. In the case of the M(^15^N)→S →M(^1^H) scheme the LLS-derived signal is very small (as compared to the thermal polarization of protons) and difficult to detect because the starting spin order is given by the low thermal polarization of the ^15^N nuclei.

In our experiments the best scheme was the M(^1^H)→S →M(^15^N) with adSLIC used as a conversion element, i.e., the ^1^H magnetization is stored in the singlet order and is detected on the ^15^N channel, even though the signal-to-noise ratio was almost 20 times greater in all spectra (this was measured in our experiments) in the case of M(^1^H)→S →M(^1^H) (the reason is that NMR detection on protons is more sensitive). Nevertheless, the presence of a large ^1^H thermal signal causes instabilities of the LLS-derived signal: compare the quality and smoothness of the curves in schemes (b) and (c) in Fig. 4.

Hence, we are able to implement all four possible scheme for generating and probing the LLS of interest. In the following subsection, we also test different schemes for sustaining the LLS and compare them to the previously used one^34^, i.e., LLS maintenance in the absence of spin-locking.

**Figure 5:**
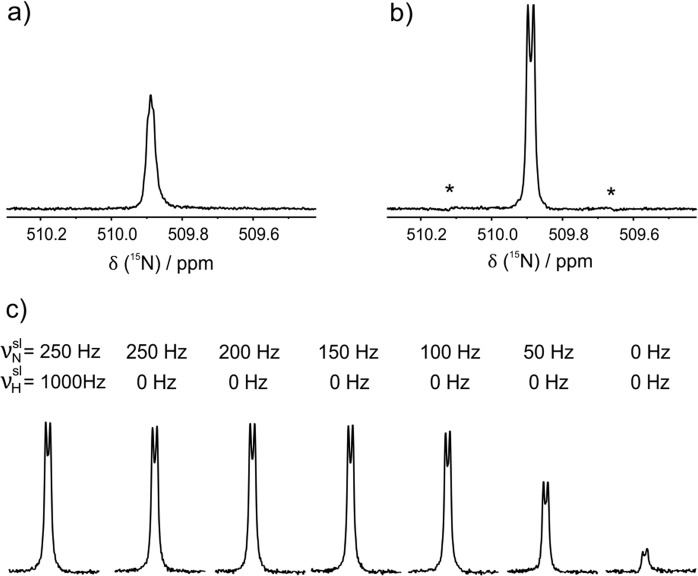
Comparison of the thermal (**a**) and LLS-derived (**b**) ^15^N-NMR spectra of *trans*-ABZ at *B*_0_ = 16.4 T. The LLS-derived signal is stronger than the thermal signal, because the singlet order was created on the ^1^H channel. Asterisks show the enhanced outer singlet-triplet transitions^52^. (**c**) Adjustment of the spin-locking scheme. Here we compare the NMR spectra coming from the long-lived spin order obtained with the singlet maintenance time of *τ* = 400 s. For M → S and S → M conversion we used adSLIC pulses: M → S was performed on the ^1^H channel and S → M was performed on the ^15^N channel. Here ${{\nu }}_{{i}}^{{sl}}$ is the strength of the spin-locking field on the corresponding channel.

The multiplet structure of the LLS-derived NMR spectrum changes significantly as compared to that of the thermal signal (compare Fig. 5a,b): the LLS-derived spectrum has the shape characteristic of a strongly coupled AB-system with a *J*-coupling of 16 Hz and an effective difference of the NMR frequencies equal to 2.1 Hz. The spectrum has 4 lines; the two outer lines are of low intensity corresponding to the “forbidden” singlet-triplet transitions^52^. The central part of the thermal spectrum is the most intense and it contains lines coming from triplet-triplet transitions in groups of states having A_2_-type sub-spectra. In the LLS-derived spectrum these transitions disappear (the LLS cannot be generated in an A_2_-system); consequently, a minimum is seen in the center of the spectrum. The same type of spectrum is found in all experiments at long *τ* delays, without and also with spin-locking, see Fig. 5c.

Spin-locking is an efficient method to increase *T*_*LLS*_ even further. Here we test different schemes, in which spin-locking is applied on the proton channel, on the nitrogen channel or on both channels simultaneously. All of these spin-locking schemes are increasing the LLS lifetime because they remove the weak magnetic non-equivalence of the ^15^N-spins by introducing proton-nitrogen decoupling.

In Fig. 5c we compare different adjustment of the spin-locking scheme: we show the ^15^N NMR spectra obtained with the same LLS maintenance time, *τ*, which is taken relative long: the *τ* = 400 s value is much longer than *T*_1_ and also about factor of three longer than *T*_*LLS*_ obtained without spin-locking. Hence, without spin-locking we obtain a relatively weak signal.

Application of spin-locking RF-fields gives rise to a significant increase of the NMR signal meaning that the LLS lifetime substantially increases. One can see that application of spin-locking fields of the strength exceeding 100 Hz on the ^15^N channel is sufficient to achieve longer *T*_*LLS*_ relaxation times. In most cases, we observe a further (though small) improvement when ^1^H spin-locking is simultaneously applied. In this case, the strength of the RF-field should be much larger than the difference in NMR frequencies of the protons in *trans-*ABZ: for *B*_0_ = 16.4 T this corresponds to 250 Hz. In this situation, all protons have the same precession frequency about the effective field in the rotating frame. Typically, a proton RF-field of the strength of 1000 Hz is sufficient.

**Figure 6:**
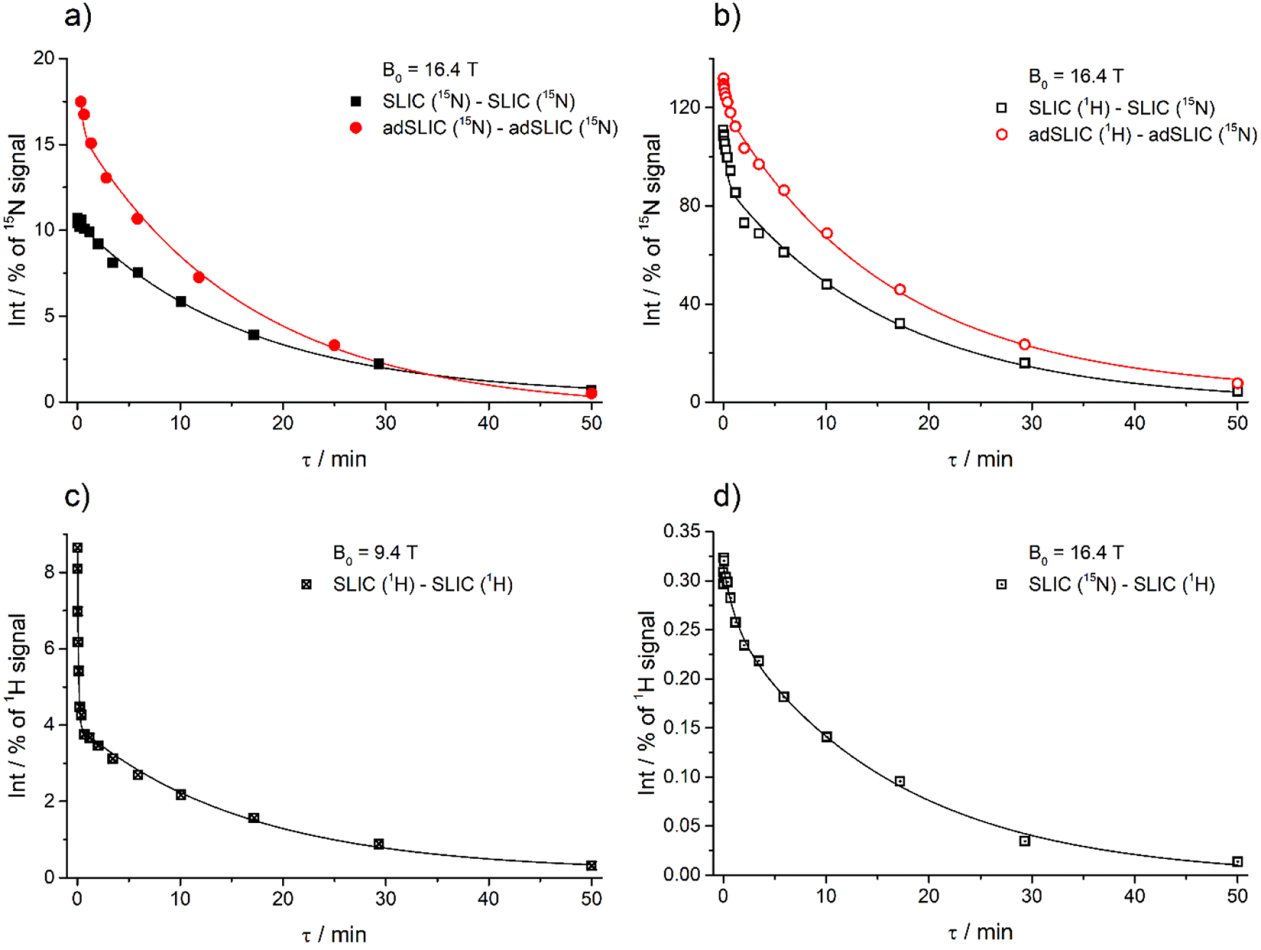
Relaxation traces obtained in experiments with strong spin-locking on the ^15^N channel (the nutation frequency ≥100 Hz) and on the ^1^H channel (the nutation frequency ≥1000 Hz) for sustaining the LLS. The M → S and S → M conversion is performed on the following channels: ^15^N-^15^N (**a**), ^1^H-^15^N (**b**), ^1^H-^1^H (**c**) and ^15^N-^1^H (**d**). Spin order conversion has been introduced using SLIC and adSLIC (the specific method is indicated for each subplot). All relaxation traces were fitted using a common lifetime *T*_*LLS*_ = 16.5 minutes of the long-lived component while the lifetime of the short living component, *T*_*f*_, was restricted to 18 s. The extracted amplitudes *A*_*LLS*_ are equal to (**a**) 10% for SLIC and 17% for adSLIC, (**b**) 90% for SLIC and 117% for adSLIC, (**c**) 4%, (**d**) 0.3%. These values were used as experimental values given in Fig. 2. We detected the same *T*_*LLS*_ at magnetic fields of 9.4 T and 16.4 T. Parameters of each individual fitting are available in the Supplementary Information for experiments #27, #28 (**a**), #36, #37 (**b**), #39 (**c**), #44 (**d**).

Figure 6 shows how the relaxation traces look like with spin-locking: one can clearly see that the lifetime of the LLS dramatically increases, compare with relaxation traces shown in Fig. 4. Here we present some typical relaxation traces; more results are shown in Supplementary Information. Thus, we can observe an increase of *T*_*LLS*_ up to ∼990 s; the best results are found when spin-locking is applied on both channels.

We want to note that the achieved *A*_*LLS*_ value, characterizing the efficiency of generating the LLS, is about 17% (for the M(^15^N)→S →M(^15^N) conversion scheme) and up to 117% (for the M(^1^H)→S →M(^15^N) scheme) when adiabatic methods are used; for resonant excitation *A*_*LLS*_ is about 10% (M(^15^N)→S →M(^15^N) scheme) and 90% (M(^1^H)→S →M(^15^N) scheme). When detection is done on the proton channel and SLIC is used as a conversion element, *A*_*LLS*_ becomes 4% (M(^1^H)→S →M(^1^H) scheme) and 0.3% (M(^15^N)→S →M(^1^H) scheme). All these values are also somewhat smaller than those shown in Fig. 4, but still in accordance with theoretical calculations.

For each scheme used for sustaining the LLS (no spin-locking, spin-locking on the nitrogen channel, spin-locking on both channels) we have performed several experiments with different spin order conversion elements. After fitting the relaxation curves we averaged the *T*_*LLS*_ values obtained for each scheme. One can see that spin-locking, which eliminates singlet-triplet leakage, increases *T*_*LLS*_ by almost a factor of 10. The ratio of the relaxation times, *T*_*LLS*_/*T*_1_, at 16.4 T becomes as large as approximately 250. Such a remarkable difference in the two relaxation times is due to the fact that at high fields *T*_*LLS*_ is immune to CSA relaxation, in contrast to *T*_1_. This observation is in agreement with previous theoretical consideration^25^ that for molecules with inversion symmetry two instantaneous local fields (induced by CSA) in areas of both ^15^N nuclear spins are the same within each molecule and thus cannot cause singlet-triplet relaxation.

## Conclusions

In this work, we elucidate the nature of the LLS in *trans*-ABZ and clearly show that it is a common property of the ^15^N spin pair as well as of the *ortho-*protons. The fact that the LLS is a common state of the nuclei of the two kinds has the following remarkable consequences. First, the LLS can be generated and detected by applying NMR pulses on either proton or nitrogen channel: in this work we inspect different schemes for magnetization-to-LLS and LLS-to-magnetization conversion. Second, the LLS lifetime can be prolonged dramatically by applying spin-locking on the ^15^N channel. These properties give a handle on efficient generation and manipulation of the LLS.

We report a detailed theoretical consideration of a six-spin system, which enables identification of the spin order conversion (M → S or S → M) pathways and calculating the conversion efficiencies. We have treated all four possible schemes of generation and detection of the LLS and compared them to the experimental results, which were also acquired for all 4 schemes. It is demonstrated experimentally that the intensity of the NMR signal coming from the LLS reaches up to 147% of the thermal ^15^N polarization when starting from the equilibrium ^1^H spin magnetization (with the adSLIC conversion method). The reason that such a strong LLS enhancement is achieved is the difference of the gyromagnetic ratios of nitrogen and proton spins, $|{\gamma }_{H}|\approx 10|{\gamma }_{N}|$, giving rise to the much higher equilibrium proton polarization. By using the ^1^H channel for detection we observed improvement of the signal-to-noise ratio (SNR), by approximately a factor of 20. However, detection on the ^1^H channel also has a disadvantage: there is a strong thermal background signal present, whereas the LLS-derived signal has the intensity less than 13% of the total signal. This requires using efficient filtering methods to remove unwanted background signals. Nevertheless, the possibility to generate and detect the LLS using pulses only on the ^1^H channel is very attractive, notably, for MRI applications ^17,50^. Hence, we have optimized the conversion stages achieving efficient M → S and S → M conversion. The experimentally achieved conversion efficiency is still lower than the maximal theoretically allowed efficiency. Nonetheless, we are able to generate and detect the LLS in a robust and reproducible way. To the best of our knowledge, except for studies of hyperpolarized spin systems, our work reports the first observation of LLS-derived NMR signals being stronger than thermal signals.

We have achieved remarkably long LLS lifetimes by optimizing the spin-locking scheme and utilizing sample confinement in a small insert for achieving high homogeneity of the *B*_0_ magnetic field and RF- field across the sample. We have observed LLS lifetimes of about 1000 s, being extra-ordinarily long for high-field experiments. We would like to stress that our study gives a unique example of an LLS existing in a complex system of 12 coupled spins in ambient conditions at high magnetic fields. The *T*_*LLS*_/*T*_1_ ratio reaches 250 at the magnetic field of 16.4 T. The factors limiting the LLS lifetimes are potentially small fluctuating interactions such as non-symmetric CSA, proton-nitrogen dipolar couplings and spin-rotation couplings. In this work, we do not analyze these factors: such an analysis would require elaborate molecular dynamics and spin dynamics simulations^48^ being out of the scope of this work.

Long-lived spin order generated with high efficiency in molecules like azobenzene is promising for various applications. First, the achieved LLS lifetimes are extremely long, allowing one to sustain the spin order for approximately 17 minutes. Second, LLS generation on ^15^N nuclei is greatly improved by using the much higher thermal magnetization of protons. Third, the molecule can be hyperpolarized by exploiting parahydrogen^36^ to increase the spin order even further paving the way to long-lived spin hyperpolarization. Last but not least, azobenzene is a photo-switchable molecule, a property which offers exciting possibilities to manipulate hyperpolarized LLSs by light excitation.

## Methods

^15^N,^15^N′-azobenzene (ABZ) was synthesized as described in ref. ^34^. The sample was prepared with a high concentration solution of 0.5 M of ABZ in CD_3_СN. It was placed in a cylindrical microcell insert (Wilmad-LabGlass, 529-E), degassed by three freeze–pump–thaw cycles and flame-sealed. The sealed microcell insert was placed into a standard 5 mm NMR tube, the NMR tube (with the insert inside) was filled with CH_3_СN. A photo of the sample is shown in Supplementary Information; this sample preparation method was used before in LLS experiments on systems of nearly equivalent spins^21,52^. By using such a sample, we keep the entire sample inside the NMR coil and get rid of unwanted effects of convection and diffusion, i.e., that the molecules do not leave the coil volume in the course of the experiment. The procedure also allows one to maintain high magnetic field homogeneity across the sample.

The NMR experiments were performed using Bruker AVANCE III spectrometers working at 400 and 700 MHz ^1^H Larmor frequency (corresponding to 9.4 T and 16.4 T magnetic fields, respectively) equipped with BBO probes for sensitive heteronuclear detection. Measurements of the singlet lifetime were performed using all four pulse schemes shown in Fig. 2 with SLIC and adSLIC used as conversion elements, see Fig. 3. Additionally, we performed experiments with the APSOC method, see Supplementary Information. A SLIC pre-saturation pulse of duration 5 *T*_1_ of ^15^N spins was applied on the ^15^N channel between successive experiments in order to remove the residual singlet order before each measurement^52^. We did not observe any influence of the temperature on the measured LLS lifetime within the range ±2 °C (all experiments were performed at around room temperature of 25 °C). Since the temperature strongly affects the position of the ^15^N line (more than a 1 Hz shift is caused by 1 °C variation) we controlled the temperature and stabilized it by applying a strong flow of dry air (500 l/h) heated to 2 °C higher than the room temperature.

SNR was measured as the maximal intensity of the signal (coming from LLS state) in the spectrum (obtained as Fourier transform of a raw FID without any apodization) divided by the root mean-square (RMS) of the intensities in the noise region. It was additionally multiplied by the square root of FID duration and divided by the square root of the number of acquisitions. This was done to compare SNR of different spectra. Note, that multiplets of *ortho-*protons are additionally split by proton-proton interactions, which were not decoupled.

The singlet order selection filter is a phase cycling method. For each point *τ* two scans were acquired: with phase of the first SLIC pulse (or adSLIC) for M → S conversion equal to *x* and then to −*x*, this was done together with cycling of the phase of the receiver (also *x* and −*x*). Such a phase cycling leads to the subtraction of all signals, which are not encoded by the first SLIC pulse. An alternative filter is the so called *T*_00_ filter based on implementation of a sequence with pulsed field gradients and RF pulses. Here, the *T*_00_ filter consisted of a gradient followed by a recovery delay and a 90° pulse with phase equal to 54.7°, then after the recovery delay another gradient was applied and followed by a third recovery delay and two 90° pulses with phases equal to 54.7° and 180°. Finally, a forth recovery delay and gradient were applied. All recovery delays were set to 5 ms. All the gradients had SINE shape and durations of 4.4 ms, 2.4 ms, and 2.0 ms, their powers were set to 10%, −10% and −15%, where 100% corresponds to a field gradient of 5.4 mT/cm.

Envelopes of constant adiabaticity pulses were calculated using a home-written Matlab code^42,53^ for an effective 2 spin system with the J-coupling equal to *J*_*NN*_ and the effective NMR frequency difference of $({}^{3}J_{HN}-{}^{4}J_{HN})$. The time profiles of the adSLIC and APSOC pulse envelopes as well the as the experimental optimization of the pulse duration are shown in Supplementary Information. The duration of the adSLIC pulse of 0.9 s was found to be sufficient to perform adiabatic passage. For the SLIC pulse, the optimal length is 0.3 s, as reported previously^34^.

In total, 44 experiments have been performed: 22 experiments without spin-locking and 22 experiments with spin-locking used for sustaining the LLS. In these experiments, we tested different conversion elements and LLS sustaining schemes. Acquisition parameters and corresponding relaxation traces are given in Supplementary Information.

## Supplementary Information

Supplementary Information.

## Data Availability

All relevant data are available from the authors.
