# Author Correction: Generating and sustaining long-lived spin states in ^15^N,^15^N′-azobenzene

**DOI:** 10.1038/s41598-020-61262-1

**Published:** 2020-03-06

**Authors:** Kirill F. Sheberstov, Hans-Martin Vieth, Herbert Zimmermann, Bogdan A. Rodin, Konstantin L. Ivanov, Alexey S. Kiryutin, Alexandra V. Yurkovskaya

**Affiliations:** 1 International Tomography Center SB RAS, Novosibirsk, 630090 Russia; 2 Helmholtz-Institut Mainz, Johannes Gutenberg-Universität, 55099 Mainz, Germany; 3 Freie Universität Berlin, 14195 Berlin, Germany; 4 Department of Biomolecular Mechanisms, Max-Planck-Institut für Medizinische Forschung, 69120 Heidelberg, Germany; 5 Novosibirsk State University, Novosibirsk, 630090 Russia

Correction to: *Scientific Reports* 10.1038/s41598-019-56734-y, published online 27 December 2019

In the Supplementary Information file originally published with this Article “Supplementary Figure 6” was omitted. This error has been corrected in the Supplementary Information file that now accompanies the Article.

