# Supplementary material for: Generating and sustaining long-lived spin states in 15N,15N′-azobenzene
Source: Sci Rep. 2019 Dec 27;9:20161. doi: 10.1038/s41598-019-56734-y (PMC6934830; doi:10.1038/s41598-019-56734-y)
Supplement: Supplementary file 1 — Supplementary Information. [file 41598_2019_56734_MOESM1_ESM.pdf]

Supplementary Information

Generating and sustaining long-lived spin states in $^{15}N,^{15}N'$-azobenzene

Authors:
Kirill F. Sheberstov,[1,2]* Hans-Martin Vieth,[1,3] Herbert Zimmermann,[4] Bogdan A. Rodin,[1,5] Konstantin L. Ivanov,[1,5] Alexey S. Kiryutin,[1,5] Alexandra V. Yurkovskaya[1,5]*

Affiliations:
[1] International Tomography Center SB RAS, Novosibirsk, 630090, Russia
[2] Helmholtz-Institut Mainz, Johannes Gutenberg-Universität, 55099 Mainz, Germany
[3] Freie Universität Berlin, 14195 Berlin, Germany
[4] Max-Planck-Institut für Medizinische Forschung, 69120 Heidelberg, Germany
[5] Novosibirsk State University, Novosibirsk, 630090, Russia

Corresponding authors:
Kirill Sheberstov, email: sheberst@uni-mainz.de
Alexandra Yurkovskaya, email: yurk@tomo.nsc.ru

Contents

1. **NMR excitation on the ^{15}N channel 2**
2. **NMR excitation on the ^{1}H channel 4**
3. **Maximal conversion efficiency 6**
4. **Numerical simulations of MSM sequences for SLIC and adSLIC 9**
5. **Experimental details 11**
6. **Results of the individual fittings of different experiments 14**
7. **References 15**

In the following sections we consider the coherent spin dynamics, which describes the creation of ^{15}N singlet order in the model spin system $AA'X_2X_2'$. We often refer to the multiple product states between nitrogen and proton states (there are a total of 64 of such product states), and importantly, some of the singlet product states of the ^{15}N pair, e.g. $|S_0^{12}, i\rangle$, are connected to the triplet states $|X_j^{12}, i\rangle$. In all such cases it is assumed, that they are connected by J-coupling interactions of the nitrogen to protons spins. These interactions govern the coherent spin dynamics responsible for creation of the ^{15}N singlet order by NMR pulse sequences. Additionally, there are fluctuating local interactions, which also connect the ^{15}N singlet manifold to the triplet one; these interactions are responsible for the relaxation of the singlet order. In this work we do not consider these interactions in the theoretical treatment.

1. NMR excitation on the ^{15}N channel

Let us first consider the situation, in which the nitrogen spin magnetization is converted into the LLS.

The spin Hamiltonian is written in the doubly rotating frame, in which the resonance frequencies of ^{1}H and ^{15}N correspond to two reference frequencies, ν_H and ν_N, and all relevant J-couplings are included:

$$\begin{aligned}\hat{\mathcal{H}}_J = J_{NN}(\hat{\mathbf{I}}_1 \cdot \hat{\mathbf{I}}_2) + J_{HH}\{(\hat{\mathbf{I}}_3 \cdot \hat{\mathbf{I}}_4) + (\hat{\mathbf{I}}_5 \cdot \hat{\mathbf{I}}_6)\} \\ + \frac{\Sigma J}{2}(\hat{I}_{1z} + \hat{I}_{2z})(\hat{I}_{3z} + \hat{I}_{4z} + \hat{I}_{5z} + \hat{I}_{6z}) \\ + \frac{\Delta J}{2}(\hat{I}_{1z} - \hat{I}_{2z})(\hat{I}_{3z} + \hat{I}_{4z} - \hat{I}_{5z} - \hat{I}_{6z})\end{aligned} \tag{1}$$

The following combinations of the *J*-couplings are introduced:

$$\Sigma J = {}^3J_{HN} + {}^4J_{HN}, \qquad \Delta J = {}^3J_{HN} - {}^4J_{HN} \tag{2}$$

Here $\hat{\mathbf{I}}_i$ is the i-th spin operator; the spins are numbered as follows: spins 1,2 are the two nitrogens, pairs of spins 3,4 and 5,6 are the *ortho*-protons of each phenyl ring (**Figure 1** in the main text of the paper). In the *J*-coupling terms we make a secular approximation for the heteronuclear interactions; hence, only z components remain.

In addition, we need to include the term that stands for RF-excitation. The key parameter is then its strength, characterized by the corresponding spin nutation frequency, $\nu_1\{^1\mathrm{H}\}$ or $\nu_1\{^{15}\mathrm{N}\}$, in the RF-field applied on the proton or nitrogen channel, respectively. For instance, the Hamiltonian of a SLIC pulse on the ^{15}N channel is given by

$$\hat{\mathcal{H}}_{RF}^N = \nu_1(\hat{I}_{1x} + \hat{I}_{2x}) \tag{3}$$

The SLIC condition requires that the nutation frequency is matched to the corresponding *J*-coupling: $\nu_1 = J$ in the tilted frame of reference, associated with the quantization axis along the x-axis of the rotating frame, and by using the singlet-triplet state vectors for each pair of spins:

$$\{SX\}_{12} = \left(\hat{\mathcal{R}}_1^y(\pi/2) + \hat{\mathcal{R}}_2^y(\pi/2)\right)\{ST\}_{12}, \quad SXSTST = \{SX\}_{12} \otimes \{ST\}_{34} \otimes \{ST\}_{56} \tag{4}$$

Here $\hat{\mathcal{R}}_i^y(\pi/2)$ is the operator introducing rotation of spins i around the y-axis by the angle of $\pi/2$. In the new basis $SXSTST$, the part of the Hamiltonian associated with the nitrogen spins is written down in a simpler way, as shown below. By $|S\rangle$ we denote the singlet state; while $|T_{+1}\rangle$, $|T_0\rangle$ and $|T_{-1}\rangle$ are the triplet states with the projection of the total spin equal to $+1$, 0 and -1, respectively.

The Hamiltonian $\hat{\mathcal{H}}_J + \hat{\mathcal{H}}_{RF}^N$ has 10 blocks, in which the singlet and triplet states of ^{15}N are connected. 8 of these blocks correspond to basis wave-functions with the z-projection of the total magnetic moment of one of the proton spin pairs being equal to ± 1, while for the other pair it is 0. In the tilted basis these blocks appear as (we also show the corresponding basis ket-vectors for the matrix elements):

$$\begin{matrix} |S_0^{12},i\rangle \\ |X_{+1}^{12},i\rangle \\ |X_0^{12},i\rangle \\ |X_{-1}^{12},i\rangle \end{matrix} \begin{pmatrix} A & -C & 0 & C \\ -C & B+\nu_1 & D & 0 \\ 0 & D & B & D \\ C & 0 & D & B-\nu_1 \end{pmatrix} \tag{5}$$

where

$$A=\frac{J_{HH}}{2}-\frac{3}{4}J_{NN}, B=\frac{J_{HH}}{2}+\frac{1}{4}J_{NN}, C=\frac{(m_{34}-m_{56})\Delta J}{2\sqrt{2}}, D=-\frac{(m_{34}+m_{56})\Sigma J}{2\sqrt{2}}$$

$$i\in\{T_{+1}^{34}S_0^{56}, T_{-1}^{34}S_0^{56}, S_0^{34}T_{+1}^{56}, T_0^{34}T_{+1}^{56}, T_{+1}^{34}T_0^{56}, T_{-1}^{34}T_0^{56}, S_0^{34}T_{-1}^{56}, T_0^{34}T_{-1}^{56}\}$$

and m_{34} is the z-projection of the total spin for protons 3 and 4, m_{56} is z-projection of the total spin for protons 5 and 6. In addition, there are two more blocks, in which the amplitude of the off-diagonal elements containing ΔJ is twice as large:

$$\begin{matrix} |S_0^{12},i\rangle \\ |X_{+1}^{12},i\rangle \\ |X_0^{12},i\rangle \\ |X_{-1}^{12},i\rangle \end{matrix} \begin{pmatrix} A & \pm C & 0 & \mp C \\ \pm C & B+\nu_1 & 0 & 0 \\ 0 & 0 & B & 0 \\ \mp C & 0 & 0 & B-\nu_1 \end{pmatrix}, \tag{6}$$

where

$$A=\frac{J_{HH}}{2}-\frac{3}{4}J_{NN}, B=\frac{J_{HH}}{2}+\frac{1}{4}J_{NN}, C=\frac{\Delta J}{\sqrt{2}}, i\in\{T_{+1}^{34}T_{-1}^{56}, T_{-1}^{34}T_{+1}^{56}\}$$

We previously determined the value of J_{HH}-coupling for *trans*-ABZ to be 2.1 Hz. However, as it can be seen from expressions (5) and (6) in all cases J_{HH} is added equally to the all diagonal elements; so it does not affect the spin dynamics of creation of the singlet state and thus can be ignored.

The appearance of the energy levels upon variation of ν_1 in the blocks of the two kinds is shown in **Figure S1**. One can see that there are well-pronounced LACs found when the matching condition $\nu_1 \approx J_{NN}$ is fulfilled. The Hamiltonian of the spin system for each of these blocks is analogous to that of a system of two strongly coupled spins-1/2[1,2]. To make this analogy mathematically correct we need to specify the effective coupling of the two levels that have an LAC. This coupling is given by the difference in the NMR frequencies of the two ^{15}N spins, being ΔJ and $2\cdot\Delta J$, in the two cases shown in **Figure S1**. By using this analogy, we can introduce methods for converting spin magnetization into long-lived spin order and back. Previously[3] we have done so by applying the APSOC method to generate the LLS in ABZ and to determine the parameters for constant adiabaticity APSOC pulses[4,5]. Here, we introduce adiabatic SLIC pulses, i.e., RF-pulses with slowly ramped amplitude so that passage through the condition $\nu_1 = J_{NN}$ is adiabatic; following previous works we introduce constant adiabaticity SLIC pulses[4]. One should note that adiabatic passage is achieved simultaneously for the blocks of the two kinds, see eqs. (5) and (6). Thus, it becomes possible to access singlet product states $|S_0^{12},i\rangle$ simultaneously in all 10 relevant blocks, which is in contrast to other methods, such as SLIC[1] and M2S[6,7]. To understand this one should consider the fact, that SLIC duration as well as the number of spin echoes in M2S methods depend on the value of the off-diagonal element connecting a particular singlet product state $|S_0^{12},i\rangle$ to a triplet product state $|X_{\pm1}^{12},i\rangle$; in this case it is given by constant C from expressions (5) and (6). The duration of SLIC and M2S cannot be tuned so that simultaneous conversion in both types of blocks is obtained.

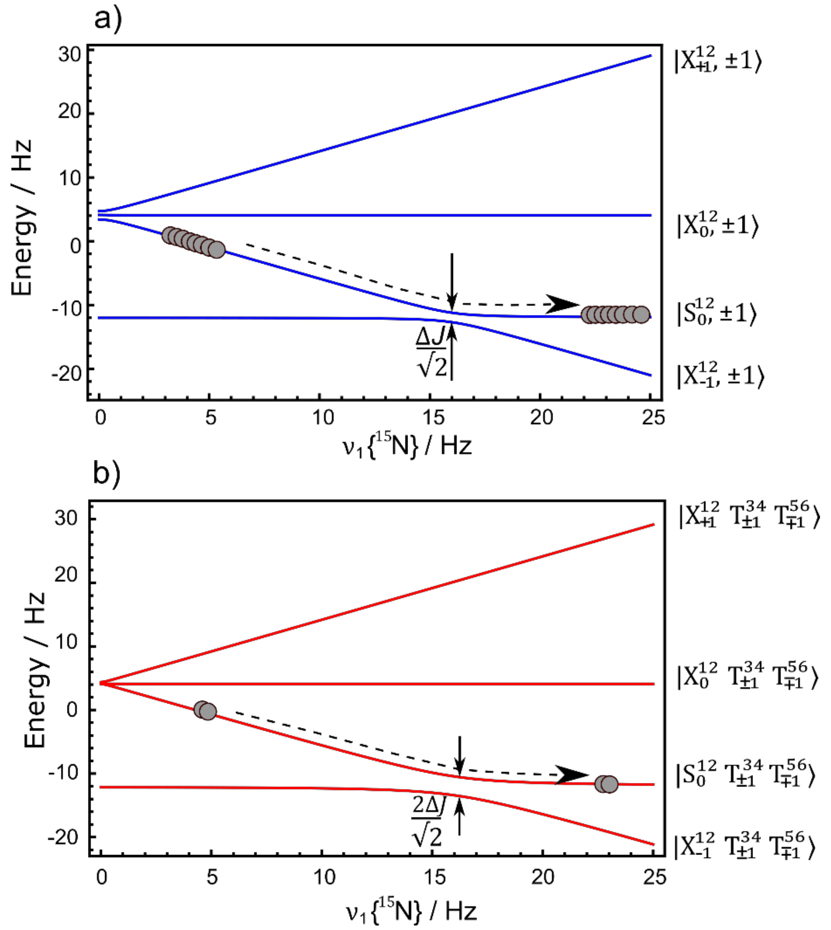

Figure S1. Energy levels as a function of the amplitude of the resonant RF-field in the ^{15}N channel, which is proportional to the nutation frequency ν_1 of the ^{15}N spins around such a field. Neglecting the shift of energy levels due to the J_{HH} coupling, we obtain that there are 8 (subplot a) and 2 (subplot b) identical blocks in the Hamiltonian. In both cases there is a level anti-crossing at $\nu_1 = J_{NN}$, which is exploited in the SLIC method. (a) For the 8 blocks with the z-projection of total angular moment of the proton spins is equal to 1 the energy splitting at the LAC is $\Delta J/\sqrt{2}$. (b) For the 2 blocks with the z-projection of the total moment of the proton spins being 0 the energy splitting at the LAC is $2\Delta J/\sqrt{2}$. In each subplot, we show the correlation of adiabatic states upon increase of ν_1, which defines the type and efficiency of spin order conversion.

2. NMR excitation on the ^{1}H channel

Now let us consider creation of the LLS using magnetization of the *ortho*-protons. In this situation, a SLIC pulse is described by the following term in the Hamiltonian:

$$\mathcal{H}_{RF}^{H} = -\nu_1\left(\hat{I}_{3x} + \hat{I}_{4x} + \hat{I}_{5x} + \hat{I}_{6x}\right) \tag{7}$$

In the presence of this term, the Hamiltonian is factorized into several blocks with connected singlet-triplet to triplet-triplet product states of the pair of ^{15}N spins. The triplet states in the tilted frame for the proton spin wave-function are defined as:

$$\left|X_l^{ij}\right\rangle = \left(\hat{\mathcal{R}}_i^y(\pi/2) + \hat{\mathcal{R}}_j^y(\pi/2)\right)\left|T_l^{ij}\right\rangle \tag{8}$$
$$l \in \{-1,0,+1\}, (i,j) \in \{(3,4),(5,6)\}$$

In this situation, there are three relevant blocks, corresponding to the following sets of basis wave-functions:

$$b1 = \{S_0^{12}X_{+1}^{34}S_0^{56},\quad S_0^{12}X_0^{34}S_0^{56},\ S_0^{12}X_{-1}^{34}S_0^{56},\quad T_0^{12}X_{+1}^{34}S_0^{56},\ T_0^{12}X_0^{34}S_0^{56},\ T_0^{12}X_{-1}^{34}S_0^{56}\},$$

$$b2 = \{S_0^{12}S_0^{34}X_{+1}^{56},\quad S_0^{12}S_0^{34}X_0^{56},\ S_0^{12}S_0^{34}X_{-1}^{56},\quad T_0^{12}S_0^{34}X_{+1}^{56},\ T_0^{12}S_0^{34}X_0^{56},\ T_0^{12}S_0^{34}X_{-1}^{56}\},$$

$$b3 = \left\{\begin{matrix} S_0^{12}\left(\frac{X_{+1}^{34}X_0^{56} + X_0^{34}X_{+1}^{56}}{\sqrt{2}}\right), & S_0^{12}\left(\frac{X_{-1}^{34}X_{+1}^{56} - X_{+1}^{34}X_{-1}^{56}}{\sqrt{2}}\right), & S_0^{12}\left(\frac{X_{-1}^{34}X_0^{56} + X_0^{34}X_{-1}^{56}}{\sqrt{2}}\right) \\ T_0^{12}\left(\frac{X_{+1}^{34}X_0^{56} + X_0^{34}X_{+1}^{56}}{\sqrt{2}}\right), & T_0^{12}\left(\frac{X_{-1}^{34}X_{+1}^{56} - X_{+1}^{34}X_{-1}^{56}}{\sqrt{2}}\right), & T_0^{12}\left(\frac{X_{-1}^{34}X_0^{56} + X_0^{34}X_{-1}^{56}}{\sqrt{2}}\right) \end{matrix}\right\}$$

For all these sets of product states, the corresponding blocks of the total Hamiltonian $\left(\widehat{H}_J + \widehat{H}_{RF}^H\right)$ are the following:

$$\begin{pmatrix} A-\nu_1 & 0 & 0 & 0 & C & 0 \\ 0 & A & 0 & C & 0 & C \\ 0 & 0 & A+\nu_1 & 0 & C & 0 \\ 0 & C & 0 & B-\nu_1 & 0 & C \\ C & 0 & C & 0 & B & 0 \\ 0 & C & 0 & C & 0 & B+\nu_1 \end{pmatrix} \tag{9}$$

where

$$A = \frac{J_{HH}}{2} - \frac{3}{4}J_{NN},\qquad B = \frac{J_{HH}}{2} + \frac{J_{NN}}{4},\qquad C = (-1)^m\frac{\Delta J}{2\sqrt{2}}$$

The sign of the off-diagonal element C is always positive for $b1$ block, always negative for $b2$ block, and for the block $b3$ the sign is positive for all transitions where T_0^{12} part changes to S_0^{12} and negative where S_0^{12} changes to T_0^{12} states.

In addition, there is one more block with a set of 10 wave-functions:

$$b4 = \left\{\begin{matrix} S_0^{12}X_{+1}^{34}X_{+1}^{56},\ S_0^{12}\left(\frac{X_{+1}^{34}X_0^{56} - X_0^{34}X_{+1}^{56}}{\sqrt{2}}\right),\ S_0^{12}\left(\frac{X_{-1}^{34}X_{+1}^{56} + X_{+1}^{34}X_{-1}^{56} - 2X_0^{34}X_0^{56}}{\sqrt{6}}\right),\ S_0^{12}\left(\frac{X_{-1}^{34}X_0^{56} - X_0^{34}X_{-1}^{56}}{\sqrt{2}}\right),\ S_0^{12}X_{-1}^{34}X_{-1}^{56} \\ T_0^{12}X_{+1}^{34}X_{+1}^{56},\ T_0^{12}\left(\frac{X_{+1}^{34}X_0^{56} - X_0^{34}X_{+1}^{56}}{\sqrt{2}}\right),\ T_0^{12}\left(\frac{X_{-1}^{34}X_{+1}^{56} + X_{+1}^{34}X_{-1}^{56} - 2X_0^{34}X_0^{56}}{\sqrt{6}}\right),\ T_0^{12}\left(\frac{X_{-1}^{34}X_0^{56} - X_0^{34}X_{-1}^{56}}{\sqrt{2}}\right),\ T_0^{12}X_{-1}^{34}X_{-1}^{56} \end{matrix}\right\}.$$

The corresponding block of the Hamiltonian is as follows:

$$\begin{pmatrix} A-2\nu_1 & 0 & 0 & 0 & 0 & 0 & C & 0 & 0 & 0 \\ 0 & A-\nu_1 & 0 & 0 & 0 & C & 0 & D & 0 & 0 \\ 0 & 0 & A & 0 & 0 & 0 & D & 0 & D & 0 \\ 0 & 0 & 0 & A+\nu_1 & 0 & 0 & 0 & D & 0 & C \\ 0 & 0 & 0 & 0 & A+2\nu_1 & 0 & 0 & 0 & C & 0 \\ 0 & C & 0 & 0 & 0 & B-2\nu_1 & 0 & 0 & 0 & 0 \\ C & 0 & D & 0 & 0 & 0 & B-\nu_1 & 0 & 0 & 0 \\ 0 & D & 0 & D & 0 & 0 & 0 & B & 0 & 0 \\ 0 & 0 & D & 0 & C & 0 & 0 & 0 & B+\nu_1 & 0 \\ 0 & 0 & 0 & C & 0 & 0 & 0 & 0 & 0 & B+2\nu_1 \end{pmatrix} \tag{10}$$

where $A = \frac{J_{HH}}{2} - \frac{3}{4}J_{NN}$, $B = \frac{J_{HH}}{2} + \frac{J_{NN}}{4}$, $C = \frac{\Delta J}{2}$, $D = \frac{\sqrt{6}}{4}\Delta J$.

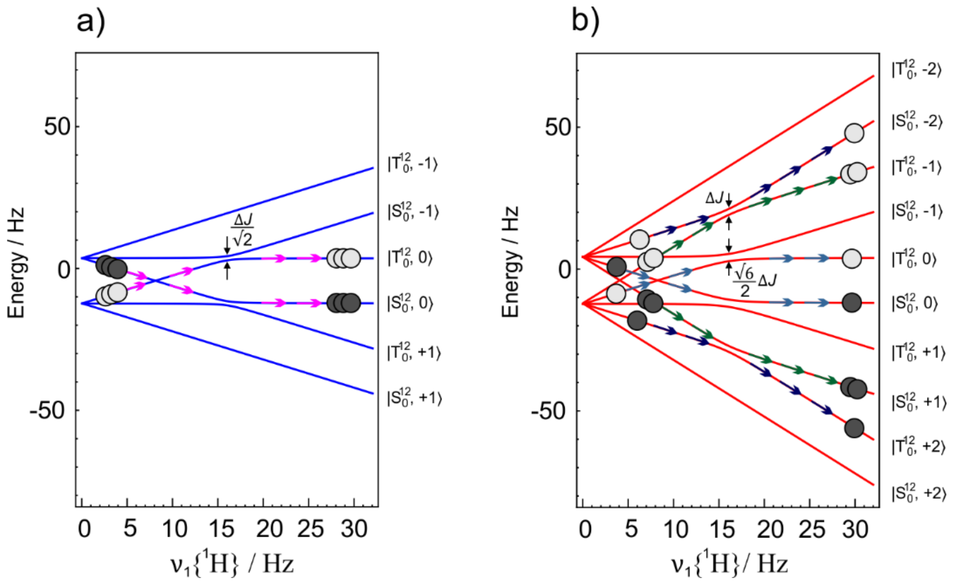

1.

Figure S2. Energy levels as a function of the spin nutation frequency around the resonant RF-field in the ^{1}H channel. As in case of **Figure S1** there is a number of LACs, in total 10 LACs. We obtain that there are 3 identical blocks with 2 LACs in each (subplot a) and a 10-level system with 4 LACs (subplot b). In (a) the minimal splitting at LACs is $\Delta J/\sqrt{2}$; in (b) the splitting is ΔJ for 2 LACs and $\sqrt{6}\Delta J/2$ for the other two LACs. Eigenstates are shown for the product states, where the first letter denotes the state of the ^{15}N pair, and the following number refers to the projection of the total spin of the *ortho*-protons along "x" axis of the ^{1}H rotated frame.

Again, it is useful to plot the energies as a function of the nutation frequency, ν_1, in this case, of the proton nutation frequency. **Figure S2** shows such plots, which allow one to identify the relevant LACs and also the correlation of adiabatic states. Again, one can see that there are several LACs found when $\nu_1 \approx J_{NN}$. The splitting of the corresponding states takes different values depending on the LAC. Note, that the same number of LACs is found when the SLIC pulse is applied on the ^{1}H and ^{15}N channel. All the LACs are always correspond to the nutation frequency $\nu_1\{^{15}N\ or\ ^1H\} = J$. Adiabatic passage over the LACs with minimal splitting ($\Delta J/\sqrt{2}$) will automatically work for all the others LACs. Consequently, the conversion elements are the same for ^{15}N and ^{1}H channels. As in the previous case, it is advantageous to use adiabatic pulses in order to exploit spin mixing at all LACs.

3. Maximal conversion efficiency

Hence, we have introduced the spin order conversion blocks, which can convert magnetization into the long-lived spin order. It is useful to assess the efficiency of the conversion for different schemes to have a reference for the experiment.

Thermal equilibrium is described by the following spin density matrix:

$$\rho_{thermal} = \frac{1}{64}\hat{E} + \frac{\Delta_{\mathrm{N}}}{64}(\hat{I}_{1z} + \hat{I}_{2z}) + \frac{\Delta_{\mathrm{H}}}{64}(\hat{I}_{3z} + \hat{I}_{4z} + \hat{I}_{5z} + \hat{I}_{6z}) \tag{11}$$

Here

$$\Delta_N = \frac{\gamma_N B_0 \hbar}{k_B T}, \Delta_H = \frac{\gamma_H B_0 \hbar}{k_B T}$$

are the Boltzmann factors of the nitrogen and proton, respectively (k_B is the Boltzmann constant and T stands for the temperature). Note, that due to the opposite signs of the gyromagnetic ratio, the "spin-down" $|\beta\rangle$ state is overpopulated for ^{15}N spins in contrast to ^{1}H spins, for which the "spin-up" $|\alpha\rangle$ state is overpopulated at thermal equilibrium. Eq. (11) is valid when the Zeeman interaction of the spins with the B_0 field is much larger than all the spin-spin *J*-couplings (high field approximation), but much smaller than the thermal energy $k_B T$ (high temperature approximation).

The degree of polarization associated with operator $\hat{P}$, as determined from a density matrix ρ, is defined as follows:[8]

$$P = \left(\hat{P}\middle|\rho\right) = \mathrm{Tr}\left\{\hat{P}^{\dagger} \cdot \rho\right\} \tag{12}$$

This kind of an expression is sometimes called "Liouville bracket" or expectation value of an operator[8]. Thus, for ^{15}N the equilibrium magnetization is calculated by taking the Liouville brackets with $\left(\hat{I}_{1z} + \hat{I}_{2z}\right)$ yielding $P^{N}_{therm} = \frac{\Delta_N}{2}$, while for protons the relevant operator is $\left(\hat{I}_{3z} + \hat{I}_{4z} + \hat{I}_{5z} + \hat{I}_{6z}\right)$ and $P^{H}_{therm} = \Delta_H$. Hence, as expected, the equilibrium magnetization of ^{1}H spins is approximately 20-fold larger than the ^{15}N magnetization: the number of protons is twice as high and their gyromagnetic ratio is *ca*. 10 times greater as compared to ^{15}N

The singlet order of a spin pair is defined as the difference between the population of the singlet state and the mean population of the triplet states of this pair:

$$\hat{P}_{SO} = \frac{1}{16}\left(\sum_{k=1}^{16} \left|S_0^{12}, k\right\rangle\left\langle S_0^{12}, k\right| - \frac{1}{3}\sum_{l=-1}^{+1}\sum_{k=1}^{16} \left|T_l^{12}, k\right\rangle\left\langle T_l^{12}, k\right|\right) = -\frac{1}{12}\left(\hat{\mathrm{I}}_1 \cdot \hat{\mathrm{I}}_2\right) \tag{13}$$

Here we calculate the maximal value of the LLS-derived NMR signal after following the sequence of events: (i) conversion of magnetization to ^{15}N singlet order, (ii) equilibration of the populations within the triplet and singlet ^{15}N manifolds, (iii) backward conversion of the LLS into magnetization.

We model the scheme with the creation and detection of the singlet order on the ^{15}N channel in the following way. At the first stage (i) the populations of the singlet product states $\left|S_0^{12}, i\right\rangle$ in the connected blocks are swapped with those of the corresponding outer triplet product states, either $\left|X_{+1}^{12}, i\right\rangle$ or $\left|X_{-1}^{12}, i\right\rangle$. In the SLIC method the $+$ or $-$ sign depends on the relative phases of the first 90-degree pulse and the SLIC pulse. For concreteness, let us assume that we populate the singlet state. In the equilibrium all the populations of the singlet product states $\left|S_0^{12}, i\right\rangle$ are *zero* with respect to populations of $\left|X_{-1}^{12}, i\right\rangle$ product states, which are overpopulated by $\frac{\Delta_N}{64}$ each. M→S conversion changes the populations of the $\left|S_0^{12}, i\right\rangle$ and $\left|X_{-1}^{12}, i\right\rangle$ product states. It is important that this conversion happens only in the blocks of the Hamiltonian presented in expressions (5) and (6), because only in these blocks the ^{15}N singlet state is connected to the triplet states by *J*-couplings. Another important thing is that this conversion is happening independently in each of these blocks. This isolation occurs because the RF-pulses do not mix blocks between each other, expressions (5) and (6) reflect this fact. Exchange of the $\left|S_0^{12}, i\right\rangle$ and $\left|X_{-1}^{12}, i\right\rangle$ populations corresponds to 100% efficiency of the M→S conversion for a particular block of the Hamiltonian. It is convenient to introduce the number n of the singlet-triplet product states in which the populations were swapped. More rigorously number n is given by:

$$n = 8\varepsilon_1 + 2\varepsilon_2 \tag{14}$$

where ε_1 and ε_2 are the efficiencies of the M→S conversion in the blocks of the first and second types respectively. **Figure S3** illustrates creation of singlet order under a SLIC pulse, applied on the ^{15}N channel. It can be seen that evolution of singlet order in the first type of blocks (expression (5)) is by a factor of two slower than in the second type (expression (6)). The maximal total population of the singlet state is achieved when the SLIC duration is set to:

$$\tau_{SLIC} = \frac{1}{\Delta J\sqrt{2}}$$

In this case singlet product states are created in 8 connected blocks of the first type (see expression (5)) and are not created at all in the 2 blocks of the second type (see expression (6)). This also agrees with previous results obtained for diphenyl acetylene[6]. So the number n is the population of the singlet state

of the ^{15}N spin pair, measured in units of $\frac{\Delta_N}{64}$ (or $\frac{\Delta_H}{64}$). As was discussed before, adiabatic methods allow to perform simultaneous conversion in all 10 blocks, so for them n equals to 10.

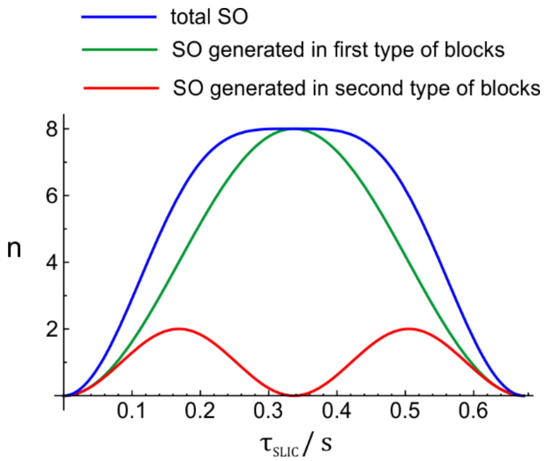

Figure S3. Creation of the ^{15}N singlet state during a SLIC pulse applied on the ^{15}N channel for a model spin system $AA'X_2X_2'$. The green trajectory shows the total population of the singlet state created in 8 blocks of the first type (expression (5)). It is given by function $8\frac{\Delta_N}{64}\sin^2\left(\pi\frac{\Delta J}{\sqrt{2}}\tau_{SLIC}\right)$. The red trajectory shows creation of the singlet state in 2 blocks of the second type (expression (6)). It is given by function $2\frac{\Delta_N}{64}\sin^2\left(\pi\frac{2\Delta J}{\sqrt{2}}\tau_{SLIC}\right)$. The blue one is given by their sum, and the same trajectory is obtained also as result of spin dynamic calculations. Note, that here the singlet state population is given in units of $\frac{\Delta_N}{64}$. Value ΔJ was set to 2.1 Hz.

Now let us continue with general considerations, which do not depend on a particular method. After M→S conversion the total population of the singlet state of the ^{15}N spins is increased by $n\frac{\Delta_N}{64}$, whereas the total population of the triplet states is decreased by $n\frac{\Delta_N}{64}$. The singlet order is calculated as the expectation value of the $\hat{P}_{SO}$ operator, given in eq. (13), here it equals to $\pm\frac{4}{3}\cdot\frac{n}{16}\cdot\frac{\Delta_N}{64}$. Again, the number n cannot exceed the number of connected blocks of the spin Hamiltonian (1), which is 10. At stage (ii) the populations of the states are averaged within all triplet manifolds as well as within the singlet manifold of the ^{15}N spin states. This process does not influence the value of the singlet order but models relaxation within these manifolds of states. At the last stage (iii) backward conversion of the singlet populations into the corresponding triplet states of the ^{15}N spins is happening. Just before the backward conversion each singlet product state $|S_0^{12}, i\rangle$ has a population of $+\frac{n}{16}\cdot\frac{\Delta_N}{64}$ and each of the triplet product states $|X_{-1}^{12}, i\rangle$ has a population of $-\frac{1}{3}\cdot\frac{n}{16}\cdot\frac{\Delta_N}{64}$. Again, only the populations of those states are swapped that belong to the connected blocks of the spin Hamiltonian.

Hence, just before NMR detection n triplet states X_{+1}^{12} or X_{-1}^{12} (depending on S→M conversion) have the populations of $+\frac{n}{16}\cdot\frac{\Delta_N}{64}$ each, while all other triplet states have populations equal to $-\frac{1}{3}\cdot\frac{n}{16}\cdot\frac{\Delta_N}{64}$. The intensity of the signal is calculated as the expectation value of the observation operator, which is $(\hat{I}_{1x} + \hat{I}_{2x})$ in this case. Thus, the amplitude of the LLS-derived signal is equal to $\frac{4}{3}\cdot\frac{n}{16}\cdot\frac{\Delta_N}{64}n$. The n^2 dependence on the number of the blocks, in which exchange between singlet and triplet populations happens, is due to the fact that the populations are swapped twice: once in the M→S stage and once again during the S→M conversion. Note, that some residual singlet order remains after the magnetization-to-singlet and singlet-to-magnetization (M→S→M) sequence.

When the singlet order is generated by applying pulses on the ^{1}H channel, redistribution of the populations between singlet and triplet states of the ^{15}N spin states is more complicated, it is graphically represented in **Figure S2**. Within all connected blocks the population of the corresponding product singlet ^{15}N states is increased while the corresponding ^{15}N product triplet states are depleted (or vice versa,

depending on the conversion type introduced by the M→S block). One should note that the populations of the ^{15}N states are exchanged with the populations of the outer triplet states of the ^{1}H spins. As a result, in all connected blocks the singlet-triplet imbalance of $2\frac{\Delta_H}{64}$ is created. The total amount of the connected blocks (and number of LACs) is the same as in the previous case, giving rise to generation of the total singlet order equal to $\pm\frac{4}{3}\cdot\frac{n}{16}\cdot\frac{\Delta_H}{64}$. This result is similar to that in the previous example; hence, the magnetization after backward conversion is equal to $\frac{4}{3}\cdot\frac{n}{16}\cdot\frac{\Delta_H}{64}n$.

These considerations can be extended to all possible M→S→M conversion schemes shown in **Figure 2** in the main text of the paper. We would like to note that exactly the same results can be obtained by considering unitary bounds for the spin dynamics[9]. However, to keep the discussion clearer, here we do not dwell on the details of such consideration.

For detection on the ^{15}N channel, the LLS-derived NMR signal is normalized to the ^{15}N magnetization and it is given by expression:

$$A_{LLS}^{N} = \frac{1}{384}\left|\frac{\gamma_i}{\gamma_N}\right| n^2, \qquad 0 \leq n \leq 10 \tag{15}$$

Here i corresponds to the type of nuclei, in which singlet order was created. For detection in the ^{1}H channel the LLS-derived NMR signal is given by expression:

$$A_{LLS}^{H} = \frac{1}{768}\left|\frac{\gamma_i}{\gamma_H}\right| n^2, \quad 0 \leq n \leq 10 \tag{16}$$

The number of the *ortho*-protons is two times greater than the number of ^{15}N spins, so normalization to the ^{1}H magnetization causes an apparent decrease of LLS population by a factor of 2 (compare eq. (15) and (16)), although it corresponds to the same polarization level. In practice, however, the scheme with detection on the ^{1}H channel should include suitable filters, to remove a strong background signal, not associated with the LLS.

4. Numerical simulations of MSM sequences for SLIC and adSLIC

To illustrate this difference in the performance of different methods we performed simulations for traditional SLIC, adiabatic SLIC with RF-excitation on the ^{15}N channel, and adiabatic SLIC with RF-excitation on the ^{1}H channel (see **Figure S4**). Calculations have been performed using the Spin Dynamica package[10].

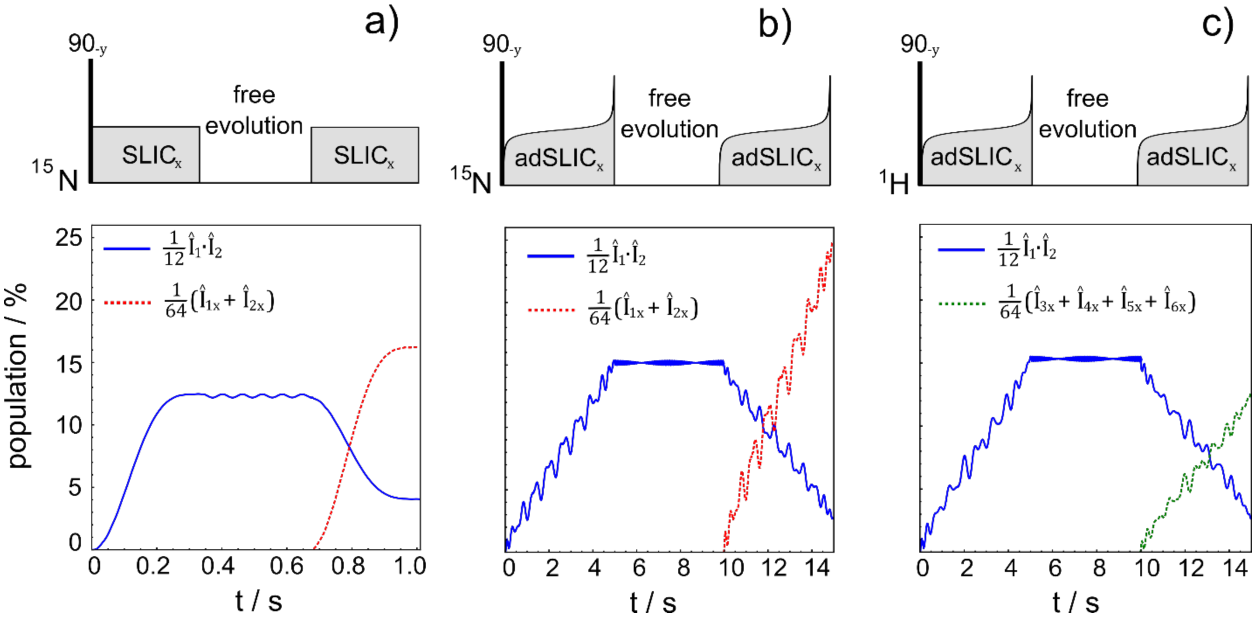

Figure S4. Evolution of the LLS population and magnetization during the M→S→M pulse sequences. Blue curves show trajectories of the normalized singlet order. Red curves show trajectories of the x-magnetization of ^{15}N spins normalized to the starting value. Green curve shows trajectory of x-magnetization of ^{1}H spins normalized to the starting value. (a) traditional SLIC on ^{15}N channel, (b) adSLIC on ^{15}N, (c) adSLIC on ^{1}H. In all cases we start with thermal populations of the spin states. Details of the calculation and normalization method are described in the text. Note, that in all cases trajectories for the starting magnetization, which evolves during the first SLIC/adSLIC pulses are not shown; their intensity always starts from 100% and depending on the method is decreasing while the singlet order is growing.

In these calculations, we considered the initial spin state, given by the following density matrix

$$\rho_0 = \frac{1}{64}(\hat{I}_{1x} + \hat{I}_{2x} + \hat{I}_{3x} + \hat{I}_{4x} + \hat{I}_{5x} + \hat{I}_{6x}) \tag{17}$$

meaning that there is only transverse magnetization present. The time dependences of magnetization and singlet order were calculated as normalized expectation values:

$$P(t) = \frac{(\hat{P}|\rho(t))}{(\hat{P}|\hat{P})} \tag{18}$$

The singlet order generated in the course of the spin evolution has been calculated as normalized expectation value of the operator $\frac{1}{12}(\hat{\mathbf{I}}_1 \cdot \hat{\mathbf{I}}_2)$. In addition, in **Figure S4** for the last S→M conversion stage we present the evolution of the magnetization of the corresponding channel as the normalized expectation value of the operator $\frac{1}{64}(\hat{I}_{1x} + \hat{I}_{2x})$ (for ^{15}N nuclei) or $\frac{1}{64}(\hat{I}_{3x} + \hat{I}_{4x} + \hat{I}_{5x} + \hat{I}_{6x})$ (for protons). We do not show the evolution of the magnetization on the first step, because it has significantly larger intensity, its evolution is basically a converse and scaled evolution of the singlet order. At the LLS maintenance stage we introduce very fast relaxation of magnetization by equilibrating the populations of all 16 singlet product states $|S_0^{12}, i\rangle$ as well as the populations of all 48 triplet product states $|T_j^{12}, i\rangle$ of the ^{15}N spin pair. This equilibration imitates such relaxation, which conserves the symmetry of ^{15}N spin states, *e.g.* mutual dipole-dipole coupling between ^{15}N spins, and the symmetric part of the CSA tensor.

Normalization introduced in eq. (18) is convenient, as it allows to present the trajectories with different norm on the same plot; also, it gives values of the final magnetization in the same manner as in eqs. (15) and (16): normalized values per equilibrium magnetization of the observation channel. We can see (**Figure S4a**) that SLIC applied on the ^{15}N channel produces final magnetization of about 16% of the starting value. A comparable value of 16.(6)% is obtained from eq. (15), when the number n is set to 8, which corresponds

to maximal conversion efficiency for SLIC. **Figure S4b** shows, that by using adSLIC pulse the final ^{15}N magnetization, coming from the LLS signal, reaches about 25%, whereas eq. (15) predicts 26% in case of $n = 10$. Finally, **Figure S4c** shows, that magnetization of protons, which originates from the LLS component reaches 12.5%, a value two times smaller than ^{15}N magnetization, because of the normalization by the total ^{1}H magnetization. It is also in agreement with eq. (16), when n is set to 10. All these amplitudes of maximal LLS signal intensities are summarized in **Figure 2** of the main text of the paper. One also can check that the singlet order calculated from the numerical values corresponds to that one, calculated in the previous section, but proper scaling should be taken into account: the values of $100 \cdot 12 \cdot \frac{4}{3} \cdot \frac{n}{16} \cdot \frac{\Delta_N}{64}$ should be compared to the SO trajectories shown in **Figure S4**.

Figure S5 illustrates the square dependence of the magnetization intensity on the efficiency of the conversion. Good agreement is observed between analytical solution of eq. (15) and numerical results from simulations with SLIC pulses of different durations. Minor deviations are caused by the fact that singlet and triplet states are not the exact eigen states due to magnetic unequivalence of the ^{15}N pair, and also by the fact that it is impossible to perform an ideal adiabatic conversion within a finite period of time.

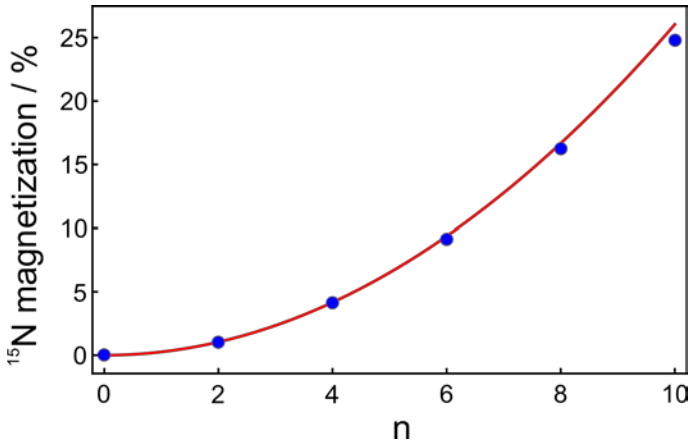

Figure S5. ^{15}N magnetization intensity (coming from LLS) as function of the number n. Blue points result from numerical simulations (identical to that one shown in **Figure S4**) for SLIC pulses of different duration. SLIC durations such that n equals to 2, 4, and 8 were calculated from **Figure S3**. Point with $n = 10$ was taken from adSLIC result (**Figure S4b**). The red curve shows the analytical solution using eq. (15): $A_{LLS}^{N} = 100\frac{1}{384}n^2$. ^{15}N magnetization is given as percentage of the equilibrium magnetization, n is given in numbers of $\frac{\Delta_N}{64}$.

So, all the main conclusions, which were made in the previous sections, are supported by **Figure S4** and **Figure S5** as well. One can see that the M→S conversion efficiency is the same for RF-excitation on the proton and nitrogen channels. In practical NMR experiments with initially thermally polarized spins, due to the 10-fold difference in the nuclear gyromagnetic ratios, this means that the LLS-derived signal is approximately 10 times stronger when the M→S block is introduced on the proton channel. These numerical simulations also show that adiabatic SLIC is more efficient than the standard SLIC method and that there is always some residual singlet order left after a 100% efficient M→S→M sequence.

5. Experimental details

Experiments of the long-lived states, which exist without spin-locking and have lifetimes of the order of minutes, are challenging. It is necessary to restrict the sample within the active volume of the detection coil. In order to achieve high stability and resolution the sample was prepared as shown in **Figure S6**: an insert (Wilmad-LabGlass, 529-E) in an NMR tube was used, filled with a solution of acetonitrile-*d*$_3$ with 1,2-^{15}N-ABZ. It was degassed by three freeze–pump–thaw cycles and sealed by flame. Protonated acetonitrile was added around the insert, to achieve good homogeneity of the magnetic susceptibility within the sample (^{1}H signals of ABZ at 700 MHz spectra had linewidths of about 0.5 Hz).

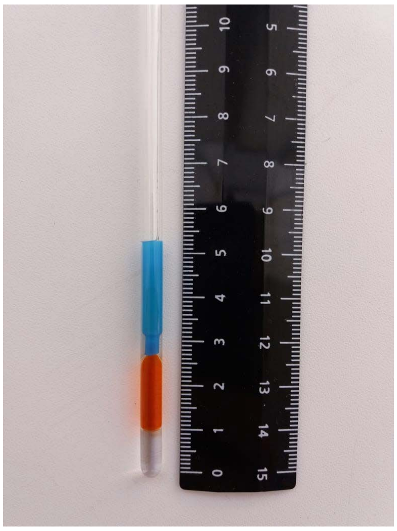

Figure S6. Sample with the insert containing a solution with *trans*-ABZ with protonated solvent around it. The position of the insert is adjusted to fit to the active volume of the detection coil.

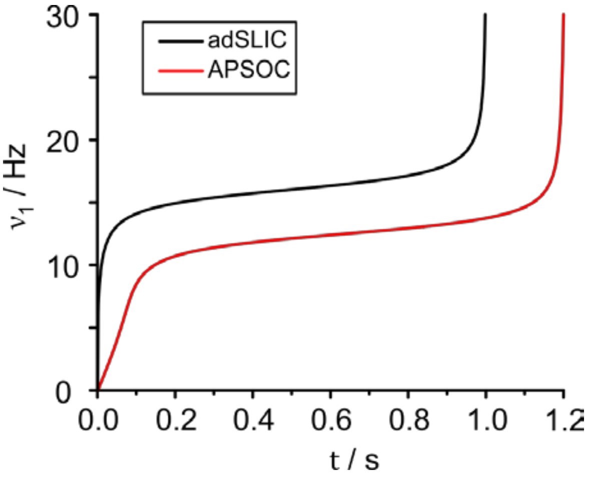

Figure S7. Time profiles of the constant adiabaticty SLIC and APSOC pulses.

Constant adiabaticity adSLIC and APSOC pulses were optimized to have constant adiabaticity using an algorithm as described in ref.4 for an effective 2 spin-1/2 system with the difference of resonance frequencies $\delta\nu = 2.1$ Hz and J-coupling equal to $J = 16$ Hz. The maximal RF-amplitude, characterized by the nutation frequency ν_1, was set to $2J$ for both methods. In the APSOC method, the adiabatic RF-pulses were applied with a frequency offset of 10 Hz relative to the resonance frequency of the NMR signal (^{1}H or ^{15}N). The resulting pulse envelopes are shown in **Figure S7**. Software for calculating constant adiabaticity profiles is available online for free[11].

Figure S8 demonstrates an optimization of the adSLIC pulse duration, aimed at achieving a high degree of adiabaticity. In this optimization, the duration of the adSLIC pulses was varied, while the τ period (LLS is sustained by using ^{15}N spin-locking) was fixed. It can be seen that starting from approximately 0.8 s of the adSLIC duration, the signal intensity reaches a plateau, corresponding to optimal performance of the conversion element (**Figure S8b**). Analogous optimization was done for the APSOC pulse in the previous study[3]; a pulse duration of 1.2 s was sufficient to achieve efficient conversion.

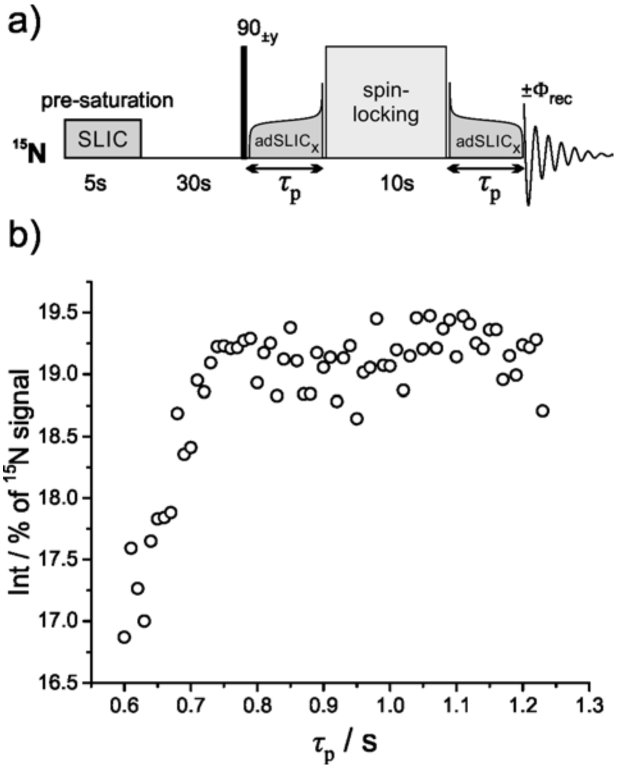

Figure S8. Optimization of adiabatic SLIC pulse duration τ_p. (a) Pulse sequence used for optimization of the pulse duration. Constant adiabaticity SLIC pulse slowly sweeps through level anti-crossing region, when nutation frequency matches J-coupling. Experiment was done at 16.4 T magnetic field. (b) Signal intensity measured as function of adSLIC pulse duration.

6. Results of the individual fittings of different experiments

Results of measuring the long-lived lifetimes in a series of experiments with different methods and conditions are summarized in **Table 1** and **Table 2**.

Table 1. Best fit parameters of the experiments without spin-locking. A bi-exponential decay was used $I(\tau) = I_0 + A_{fast}\exp\{-\tau/T_f\} + A_{LLS}\exp\{-\tau/T_{LLS}\}$. Gradient filter T_{00} was used in some experiments the same as in ref.[12] (otherwise the singlet order selection filter was always used). It is denoted at which channel the hard pulses were applied, and in some cases the filter was repeated twice.

# of experiment	scheme	method	field, T	T_{00} filter (1)	I_0, %	A_{fast}, %	T_f, s	A_{LLS}, %	T_{LLS}, s
1	^{15}N to ^{15}N	SLIC	16.4	(^{1}H, ^{15}N)	0.2 ± 0.1	4 ± 1	1.1 ± 0.2	11.3 ± 0.1	139 ± 5
2	^{15}N to ^{15}N	SLIC	16.4	(^{1}H, ^{15}N)	0.0 ± 0.2	< 0.1	-	11.3 ± 0.2	149 ± 6
3	^{15}N to ^{15}N	SLIC	16.4	(^{1}H)	-0.1 ± 0.2	2 ± 0.4	2.4 ± 0.8	11.6 ± 0.2	140 ± 7
4	^{15}N to ^{15}N	SLIC	16.4	(^{15}N)	-0.3 ± 0.1	1.6 ± 0.15	4.5 ± 1	11.4 ± 0.1	154 ± 6
5	^{15}N to ^{15}N	adSLIC	16.4	-	0.6 ± 0.2	5.1 ± 0.2	5.9 ± 0.7	17.8 ± 0.2	137 ± 5
6	^{15}N to ^{15}N	adSLIC	16.4	-	0.1 ± 0.2	5.1 ± 0.2	5.8 ± 0.7	18.4 ± 0.2	147 ± 6
7	^{15}N to ^{15}N	adSLIC	16.4	-	0.5 ± 0.2	5.3 ± 0.3	5.6 ± 0.7	18.4 ± 0.2	139 ± 6
8	^{15}N to ^{15}N	adSLIC	16.4	(^{15}N) x2	0.8 ± 0.2	4.1 ± 0.2	6.5 ± 0.9	17.8 ± 0.2	135 ± 5
9	^{15}N to ^{15}N	adSLIC	16.4	(^{1}H) x2	1.2 ± 0.2	1.7 ± 0.2	7 ± 2	16.6 ± 0.2	145 ± 5
10	^{15}N to ^{15}N	adSLIC	16.4	(^{1}H, ^{15}N) x2	0.2 ± 0.2	< 0.1	-	19.0 ± 0.2	144 ± 3
11	^{15}N to ^{15}N	APSOC	16.4	-	0.6 ± 0.5	8 ± 1	3.6 ± 0.7	17.6 ± 0.5	130 ± 11
12	^{1}H to ^{15}N	adSLIC	16.4	-	3.8 ± 1.6	22 ± 2	6 ± 1	143 ± 2	137 ± 5
13	^{1}H to ^{15}N	adSLIC	16.4	-	3.9 ± 1.5	22 ± 2	7 ± 1	146 ± 2	137 ± 5
14	^{1}H to ^{15}N	adSLIC	16.4	-	3.3 ± 1	22 ± 1	8 ± 1	147 ± 1	138 ± 3
15	^{1}H to ^{15}N	SLIC	16.4	(^{1}H, ^{15}N)	1.9 ± 0.7	19 ± 1	3.5 ± 0.5	101 ± 1	123 ± 3
16	^{1}H to ^{15}N	adSLIC	9.4	(^{1}H, ^{15}N) x2	2.3 ± 1	< 1	-	136 ± 1	104 ± 3
17	^{1}H to ^{15}N	adSLIC	9.4	(^{1}H, ^{15}N) x2	-0.1 ± 0.5	4 ± 1	9 ± 5	159 ± 1	114 ± 2
18	^{1}H to ^{1}H	SLIC	16.4	(^{1}H, ^{15}N)	0.1 ± 0.1	1.8 ± 0.1	5.3 ± 1	5.6 ± 0.1	109 ± 6
19	^{1}H to ^{1}H	SLIC	16.4	(^{1}H)	0.1 ± 0.1	2.3 ± 0.3	2.8 ± 0.7	4.9 ± 0.2	113 ± 11
20	^{1}H to ^{1}H	SLIC	9.4	(^{1}H)	-0.1 ± 0.2	3.4 ± 0.3	2.8 ± 0.6	5.8 ± 0.2	114 ± 12
21	^{1}H to ^{1}H	SLIC	9.4	(^{1}H)	-0.1 ± 0.1	3.8 ± 0.4	2.1 ± 0.3	6.2 ± 0.1	107 ± 7
22	^{15}N to ^{1}H	SLIC	16.4	(^{1}H, ^{15}N)	0.03 ± 0.01	1.3 ± 1.8	0.3 ± 0.1	0.25 ± 0.01	116 ± 11

Table 2. Best fit parameters of the experiments with spin-locking. A bi-exponential decay was used: $I(\tau) = I_0 + A_{fast}\exp\{-\tau/T_f\} + A_{LLS}\exp\{-\tau/T_{LLS}\}$.

# of experiment	scheme	method	field, T	Spin-locking ^{15}N, Hz	Spin-locking ^{1}H, Hz	I_0, %	A_{fast}, %	T_f, s	A_{LLS}, %	T_{LLS}, s
23	^{15}N to ^{15}N	APSOC	9.4	100	500	0.1 ± 0.4	3.9 ± 0.4	4.9 ± 1.1	18.0 ± 0.4	1007 ± 59
24	^{15}N to ^{15}N	APSOC	9.4	100	-	0.5 ± 0.3	36 ± 0.3	7.6 ± 0.2	17.7 ± 0.3	718 ± 33
25	^{15}N to ^{15}N	APSOC	9.4	-	500	0.3 ± 0.4	34 ± 3	1.3 ± 0.1	16.7 ± 0.4	555 ± 41
26	^{15}N to ^{15}N	SLIC	9.4	100	-	0.0 ± 0.3	7.7 ± 0.3	6.5 ± 0.6	9.7 ± 0.3	779 ± 68
27	^{15}N to ^{15}N	adSLIC	16.4	250	1000	-0.3 ± 0.2	3.7 ± 0.3	76 ± 15	15.3 ± 0.3	1022 ± 57
28	^{15}N to ^{15}N	SLIC	16.4	250	1000	0.4 ± 0.3	< 0.1	-	10.2 ± 0.3	970 ± 57
29	^{15}N to ^{15}N	SLIC	16.4	250	-	0.9 ± 0.3	2.4 ± 0.3	4.3 ± 1	10.1 ± 0.3	769 ± 54
30	^{15}N to ^{15}N	SLIC	16.4	-	1000	0.0 ± 0.1	0.1 ± 0.2	5.3 ± 2.7	11.3 ± 0.3	291 ± 9
31	^{1}H to ^{15}N	APSOC	9.4	100	1500	2 ± 2	34 ± 1.7	5.8 ± 0.7	88 ± 2	1120 ± 70
32	^{1}H to ^{15}N	APSOC	9.4	100	500	-0.6 ± 2	39 ± 2	5.9 ± 0.6	88 ± 2	1086 ± 65
33	^{1}H to ^{15}N	APSOC	9.4	100	-	0 ± 1.5	38 ± 2	5.3 ± 0.6	91 ± 1.5	715 ± 34
34	^{1}H to ^{15}N	APSOC	9.4	100	-	0.3 ± 1.6	41.5 ± 2	6.3 ± 0.6	88 ± 1.6	862 ± 44
35	^{1}H to ^{15}N	APSOC	9.4	-	500	-0.6 ± 1.4	32 ± 2	6.7 ± 1	88 ± 1.6	599 ± 32
36	^{1}H to ^{15}N	adSLIC	16.4	250	1000	-1.3± 1.9	14± 1.3	40 ± 9	118 ± 1.6	1140 ± 53
37	^{1}H to ^{15}N	SLIC	16.4	250	1000	-2.8 ± 3	30 ± 2.5	60 ± 9.5	84 ± 2.4	1198 ± 126
38	^{1}H to ^{15}N	SLIC	16.4	250	1000	-2.7 ± 3.5	31 ± 3	61 ± 11	82 ± 2.9	1217 ± 154
39	^{1}H to ^{1}H	SLIC	9.4	100	1500	0.6 ± 0.4	5.5 ± 0.4	5.9 ± 0.5	3.3 ± 0.4	886 ± 127
40	^{1}H to ^{1}H	SLIC	9.4	100	500	0.5 ± 0.4	3.3 ± 0.4	9.7 ± 1.5	4.1 ± 0.4	885 ± 132
41	^{1}H to ^{1}H	adSLIC	9.4	200	1000	-0.1 ± 0.1	3.3 ± 0.1	16 ± 1	4.3 ± 0.1	1092 ± 55
42	^{1}H to ^{1}H	APSOC	9.4	100	500	-0.3 ± 0.2	8.3 ± 0.2	11 ± 0.6	5.5 ± 0.2	985 ± 108
43	^{1}H to ^{1}H	APSOC	9.4	100	500	0.1 ± 0.3	7.9 ± 0.2	11 ± 0.8	5.1 ± 0.3	1041 ± 163
44	^{15}N to ^{1}H	SLIC	16.4	250	1000	0.0 ± 0.01	0.05 ± 0.02	71 ± 40	0.3 ± 0.02	991 ± 180

7. References

1 DeVience, S. J., Walsworth, R. L. & Rosen, M. S. Preparation of Nuclear Spin Singlet States Using Spin-Lock Induced Crossing. *Phys. Rev. Lett.* **111**, 173002, doi:10.1103/PhysRevLett.111.173002 (2013).

2 Pravdivtsev, A. N., Kiryutin, A. S., Yurkovskaya, A. V., Vieth, H.-M. & Ivanov, K. L. Robust conversion of singlet spin order in coupled spin-1/2 pairs by adiabatically ramped RF-fields. *J. Magn. Reson.* **273**, 56-64, doi:10.1016/j.jmr.2016.10.003 (2016).

3 Sheberstov, K. F. *et al.* Cis versus trans-Azobenzene: precise determination of NMR parameters and analysis of long-lived states of ^{15}N spin pairs. *Appl. Magn. Reson.* **49**, 293-307, doi:10.1007/s00723-017-0968-8 (2018).

4 Rodin, B. A. *et al.* Constant-adiabaticity RF-pulses for generating long-lived singlet spin states in NMR. *J. Chem. Phys.* **150**, 064201, doi:10.1063/1.5079436 (2019).

5 Rodin, B. A. *et al.* Using optimal control methods with constraints to generate singlet states in NMR. *J. Magn. Reson.* **291**, 14-22, doi:10.1016/j.jmr.2018.03.005 (2018).

6 Feng, Y., Theis, T., Wu, T. L., Claytor, K. & Warren, W. S. Long-lived polarization protected by symmetry. *J. Chem. Phys.* **141**, 134307, doi:10.1063/1.4896895 (2014).

7 Tayler, M. C. D. & Levitt, M. H. Singlet nuclear magnetic resonance of nearly-equivalent spins. *Phys. Chem. Chem. Phys.* **13**, 5556-5560, doi:10.1039/C0cp02293d (2011).

8 Jeener, J. Superoperators in Magnetic-Resonance. *Adv. Magn. Res.* **10**, 1-51, doi:10.1016/B978-0-12-025510-8.50006-1 (1982).

9 Levitt, M. H. Symmetry constraints on spin dynamics: Application to hyperpolarized NMR. *J. Magn. Reson.* **262**, 91-99, doi:10.1016/j.jmr.2015.08.021 (2016).

10 Bengs, C. & Levitt, M. H. SpinDynamica: Symbolic and numerical magnetic resonance in a Mathematica environment. *Magn. Reson. Chem.* **56**, 374-414, doi:10.1002/mrc.4642 (2018).

11 *Web page for adiabatic pulse generation and APSOC optimization*, <http://www.tomo.nsc.ru/en/nmr/>

12 Tayler, M. C. D. & Levitt, M. H. Accessing Long-Lived Nuclear Spin Order by Isotope-Induced Symmetry Breaking. *J. Am. Chem. Soc.* **135**, 2120-2123, doi:10.1021/Ja312227h (2013).
